# Regulating photoreactivity in a polymorphic bi-component solid through large synthons

**DOI:** 10.1038/s42004-025-01527-w

**Published:** 2025-04-30

**Authors:** Mollah Rohan Ahsan, Arijit Mukherjee

**Affiliations:** Department of Chemistry, Birla Institute of Technology and Science, Pilani, Hyderabad Campus, Medchal District, Telangana India

**Keywords:** Crystal engineering, Self-assembly, Photochemistry

## Abstract

Polymorphism in bi-component crystals has received immense attention in recent times due to their potential in property engineering. Two polymorphs of a functional bi-component molecular solid are derived using Long-range Synthon Aufbau Modules (LSAM) by tuning the nature and position of substituents. The two polymorphs show distinct photochemical properties; **Form I** undergoes partial *cis-trans* isomerization, and **Form II** sustains a [2 + 2] photodimerization. The polymorphic forms also exhibit different photomechanical behaviors. While **Form I** shows differing photomechanical responses upon UV irradiation based on crystal morphology, **Form II** undergoes a solid-to-liquid transition upon photo-irradiation.

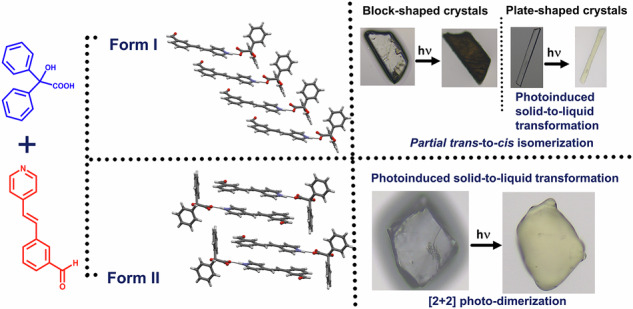

## Abstract (web summary)

Polymorphism in bi-component crystals offers promising avenues for property engineering. Here, the authors utilize long-range synthon Aufbau modules to derive two polymorphs with unique photoreactivity and photomechanical properties.

## Introduction

Design and synthesis of functional crystalline solids have become one of the most attractive research areas in chemistry and material science^1,2^. Crystal engineering, a subject primarily dedicated to the rational design of molecular solids^3^, has shown immense potential in designing new solid materials having applications in a vast range of diverse fields, including pharmaceuticals^4^, flexible crystals^5,6^, optoelectronic materials^7^, piezoelectric crystals^8^, and photoreactivity^9–11^, among others. One of the significant ways crystal engineering principles were applied to the above applications is through the design and synthesis of multicomponent solids^12^. Although the first report of such solids dates back to the 1800s^13^, it is in the past three decades that the design of these solids is perceived systematically, and novel synthetic strategies have been coined^14^. With the advancement of the subject of crystal engineering, structural variability in multi-component systems is analyzed more systematically, especially in the context of a structural landscape^15^. Stoichiometric multicomponent solids are generally classified into two major categories: (i) cocrystals and (ii) organic salts, while the difference among these can sometimes be dictated by complete or partial transfer of protons between donors (D) and acceptors (A) in different compounds^16^. The discovery of polymorphism in both these scenarios is important from a structural and property engineering viewpoint.

**Scheme 1:**
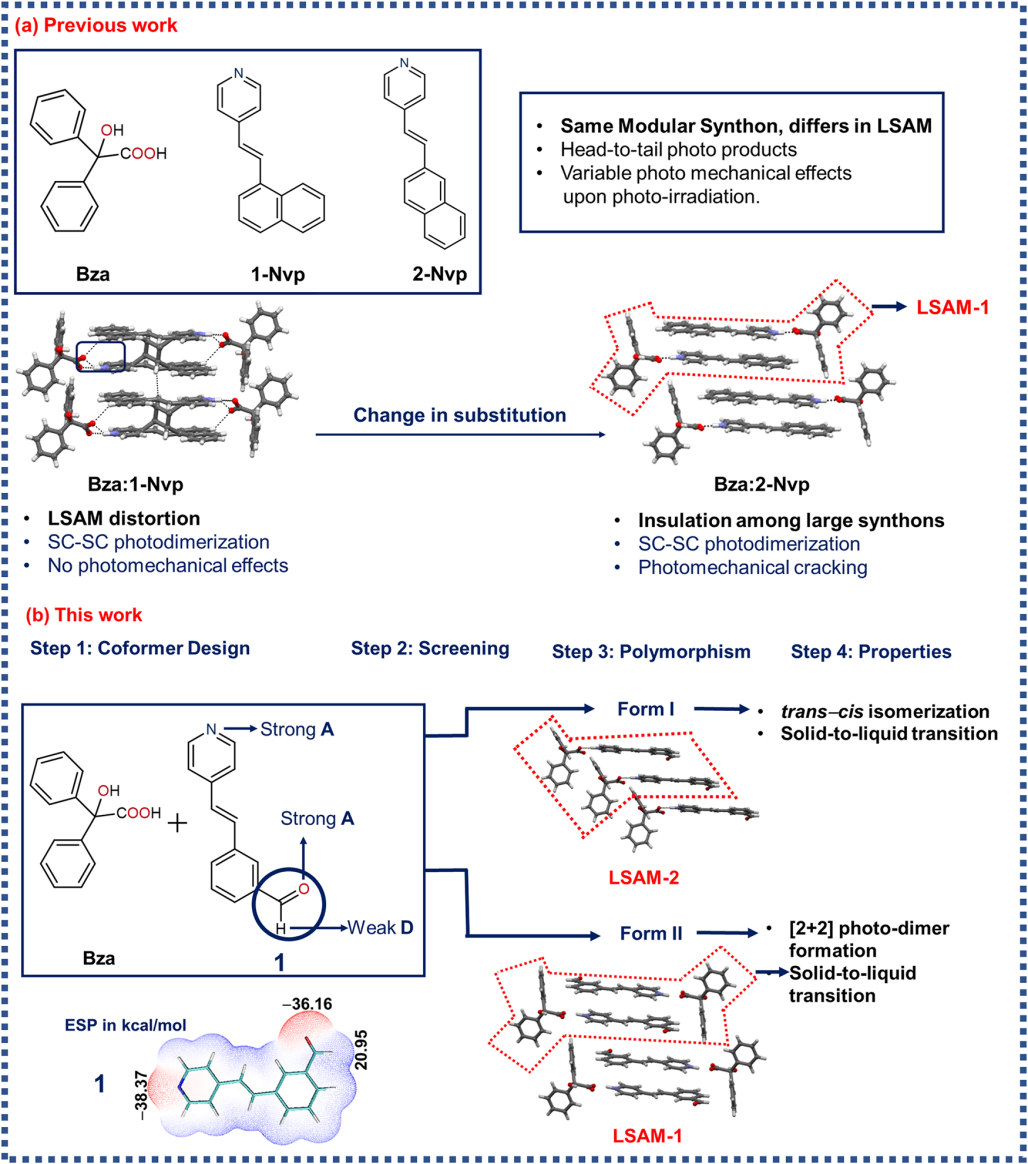
Searching for polymorphs in a bicomponent solid: a crystal engineering approach to discover polymorphs with different Long-Range Synthon Aufbau Modules (LSAM). The red-dotted box represents the LSAMs. **a** Previous work done on related **Bza-**based bi-component systems, **b** key features of the current work.

Our primary interest lies in the design of multicomponent solids based on benzilic acid (**Bza**) to direct [2 + 2] homodimerization in organic solids (Scheme 1). **Bza**, often perceived in the context of Benzil-Benzilic acid rearrangement^17^, was exploited recently as a template to induce photoreactivity in the otherwise unreactive stilbazole derivatives^18^. This approach, on the one hand, has shown synthon robustness and large synthon modularity; on the other, it has led to intriguing photochemical and photomechanical properties^18–20^. The modular large synthon, often called Long-Range Synthon Aufbau Module (LSAM)^21^, derives from both chemical factors, such as strong hydrogen bonding, π···π stacking interactions and geometrical factors, such as inversion center. As most of the cocrystals designed in Bza-stilbazole derivatives show the presence of a single LSAM (LSAM-1) with subtle variations, it indicates a regular Aufbau process^3^. Since LSAMs are modular and bigger units than typical supramolecular synthons, variability in the packing of the designed solids should also be minimal. This is reflected in the discovery of only one polymorphic system in Bza-stilbazole class of bi-component solids, despite several efforts^20^. Even in the said system, the LSAM-1 remains intact, and the polymorphs are packed in *P*$\bar{1}$ and *P*2_1_/*c*, following Kitaigordskii’s dictum for packing of inversion-related molecules^22^, leading to similar photochemical and photomechanical properties upon irradiation. It is rare, therefore, to find polymorphs in this class and other photoreactive bi-component solids where polymorphs exhibit distinctly different photochemical properties. A clear research gap, therefore, exists towards the isolation of polymorphic bi-component systems with distinctly different photochemical and photomechanical responses. Structurally, this scenario generally arises due to the similarity of synthons in different polymorphs. Since synthon polymorphism is extremely rare and needs certain restrictions on the nature and positions of functional groups^23^, design elements that lead to different LSAMs may be sought to overcome this problem; we were especially interested in exploring the ways to access alternative large synthon possibilities (and resultant packing) in **Bza**-based bi-component solids. In a recent study based on a multicomponent solid between Bza and ((*E*)-4-(2-(naphthalene-1-yl)vinyl)pyridine) (henceforth **1-Nvp**)^19^, we showed that LSAM distortion can be a result of the structural interference of neighboring LSAMs and changing the molecular substitution by replacing **1-Nvp** with **2-Nvp** led to structural insulation among the LSAMs (Scheme 1a) resulting in a slow cracking upon photo-irradiation^20^. Taking a clue from this exercise, in hindsight, we thought of designing a stilbazole derivative with a strong hydrogen bond acceptor and placing it in an orthogonal direction to the long molecular axis. Following the idea of maximal use of strong H-bond acceptors and donors^24,25^, a different large synthon could be envisaged (Scheme 1b) if the multi-component solid is formed at all.

In this context, we chose to synthesize ((*E*)-3-(2-(pyridin-4-yl)vinyl) benzaldehyde) (**1)** to co-crystallize with **Bza**. The electrostatic potential (ESP) calculations on **1** show it has two acceptor sites and one donor site. The acceptor sites are located on N (*V*_max_ = −38.37 kcal/mol, associated with N lone pair) and aldehydic O ((*V*_max_ = −36.16 kcal/mol, associated with O lone pair). The donor site is located on aldehydic H (*V*_max _= 20.95 kcal/mal). It may be instructive to note that both the acceptor sites are extremely strong, with pyridine-N slightly more basic than aldehydic-O (Scheme 1). Despite several trials, we could not obtain single crystals of **1**. From the ^1^H NMR spectra of the dissolved powder samples before and after photoirradiation, **1** seemed unreactive (Supplementary Method, Supplementary Fig. S19).

## Results and discussion

### Cocrystallization of Bza with 1

**Fig. 1:**
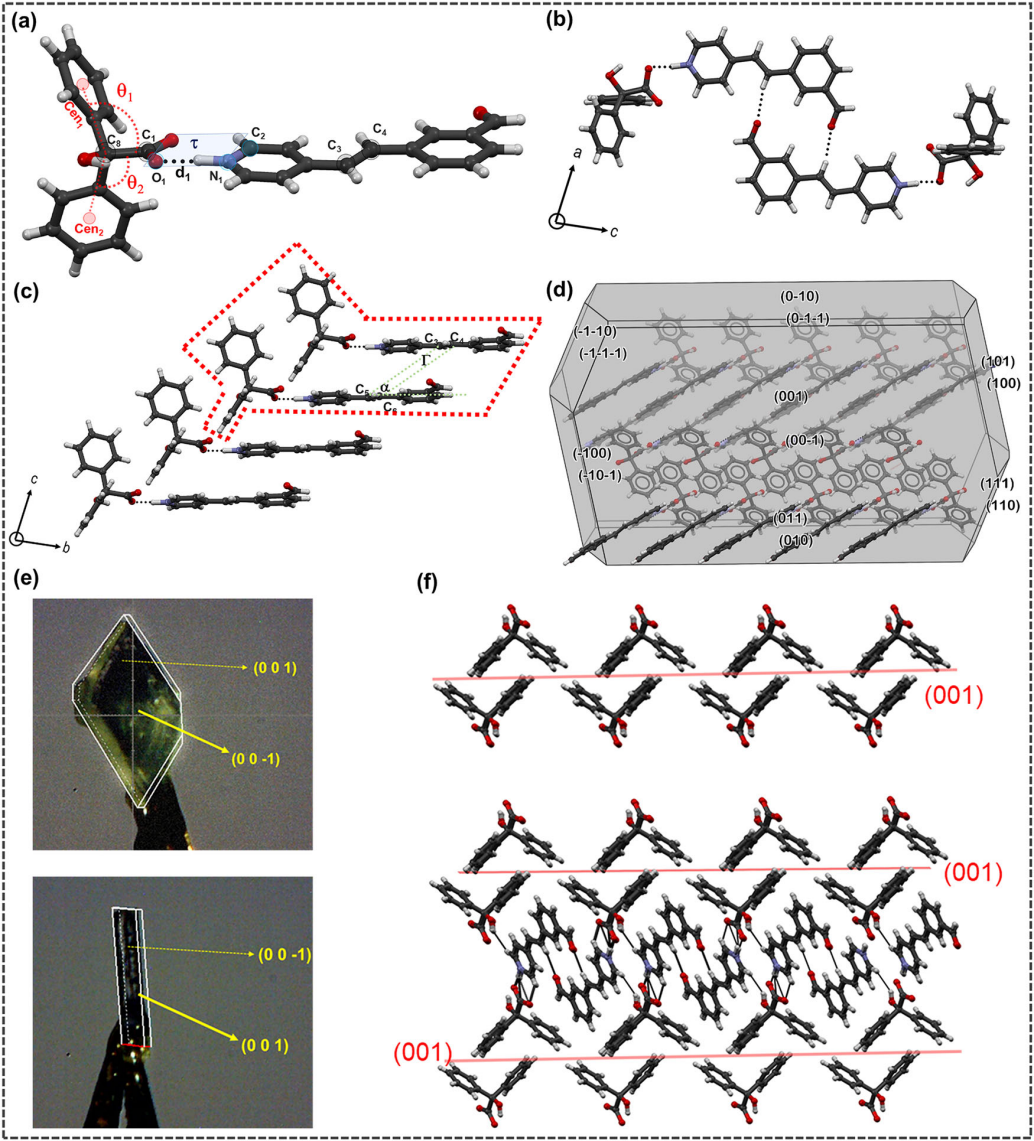
Packing of Bza-1-Form 1. **a** Primary synthon formed between **Bza** and **1**. **b** crystal packing along *b*-axis, and the C−H···O interaction among **1**. **c** crystal packing along *a*-axis, showing the *h-h* orientation of **1**. LSAM 2 is marked with a red dotted line. **d** Calculated morphology of **Bza-1-Form I**. **e** face indexing images of **Bza**-**1**-**Form I** (block and plate-shaped crystals). **f** Molecular orientation around (001) plane. Geometrical parameters: O−N bond distance (d_1_), the torsional angle between the carboxylate and pyridinium synthon (*τ* = C_1_−O_1_−N_1_−C_2_), C=C distance (**d**_**1**_ = C_3_−C_5_), the angle between the C=C bonds (**α** =$<$ C_3_−C_5_−C_6_), and the torsional angle (Γ = C_3_−C_4_−C_5_−C_6_) between the C=C bonds.

When **Bza** and **1** were cocrystallized in a (1:1) molar ratio from ethanol, block-shaped crystals were obtained, crystallizing in *P*$\bar{1}\,$(Supplementary data 1, Suppementary Note 1). **Bza** interacts with **1** through a charge-assisted O^-^···H$-$N^+^ (1.54(2) Å, 175.7(19)°) hydrogen bonding, and the torsional angle (*τ*) around the respective carboxylate-pyridinium synthon is ∼5.0(1)°. The phenyl rings of the **Bza** molecule are oriented almost orthogonally (*θ*_1 _= 105.33°, *θ*_2 _= 108.24°) with respect to the **1**. Despite the formation of an O^−^···H$-$N^+^ synthon, this structure sustains through LSAM-2 where primary synthons are oriented in a *head-to-head (h-h)* fashion. The primary reason behind this may be traced through a C−H···O (2.445 Å, 159.70 °) between aldehydic-O and vinylic- H in the neighboring LSAMs (Fig. 1b). The distance between two molecules of **1** lies around 6.625(2) Å, well beyond the permissible distance (∼4.1 Å) for [2 + 2] photo-dimer formation. This also indicates a weaker π···π interaction, leaving a possibility of isomerization in the resultant solids after exposure to UV irradiation.

An exhaustive polymorph screening was conducted in search of alternative forms. This led to **Form I** in all the crystallization experiments (Supplementary Method, Supplementary Figs. S4-S12), primarily indicating other forms may be difficult to obtain under ambient conditions, in our hand. Details of crystallization experiments are provided in Supplementary Method and Supplementary Note-2.

Two types of crystal morphology (plate and block) were observed for **Bza-1-Form I**. Face indexing of both types of crystals shows that the dominant face is (001), aligned with the *c*-axis. Further calculation based on the Bravais-Friedel-Donnay-Harker (BFDH) procedure (Fig. 1d) agrees well with the experimental observations. The molecular arrangement on (001) shows that it consists of weak interactions among **Bza** molecules, justifying this to be the slowest-growing face. The molecules of **1** are positioned diagonally with respect to the (100) plane. Primary analysis of the packing also suggests that the thickness of these crystals may be controlled by hydrogen bonding between **Bza** and **1** molecules (Fig. 1f).

### Polymorph screening in the presence of a competitive equilibrium

**Fig. 2:**
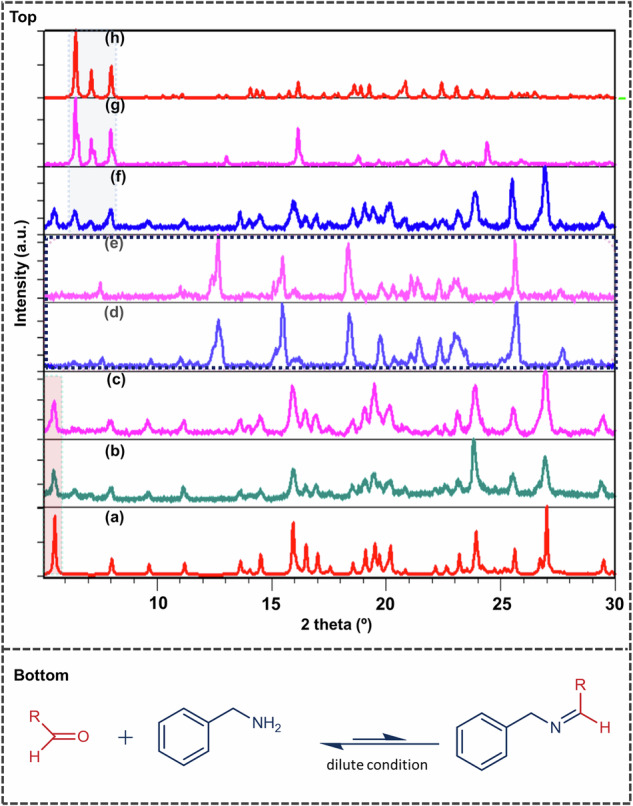
Polymorph screening in competitive equilibrium. **Top-**Crystallization outcome of **Bza-1** mixture from varying % of benzylamine/ MeOH solution. **a** The simulated pattern of Bza-1-Form I. **b** solution crystallization outcome from 2% of **2** in methanol solution. It gives rise to **Bza-1-Form I**. **c** Solution crystallization outcome from 6% of **2** in methanol solution. It gives rise to **Bza-1-Form I**, **d** solution crystallization outcome from 8% of **2** in methanol solution. **e** Solution crystallization outcome from 12% of **2** in methanol solution, same PXRD pattern as observed in (**d**); **f** Solution crystallization outcome from 20% of **2** in methanol solution: gives rise to a mixture of **Bza-1-Form I** and **Bza-2** salt, **g** solution crystallization outcome from 70% of **2** in methanol solution: gives rise to **Bza-2** salt, **h** simulated PXRD pattern of **Bza-2** salt. **Bottom--**a proposed scheme for imine exchange.

As mentioned in the previous section, a robust polymorph screening procedure led to **Form I** in all the cases. In such a scenario, one may wonder if accessing alternative forms, specifically a form with LSAM-1 (Scheme 1), is at all possible. In 2005, Davey showed that a structure-solution link can exist in some polymorphic systems^26^, and therefore, tuning the nucleation step can be a key step in future crystal engineering exercises^27^. Later, in obtaining single crystals of covalent organic frameworks, Yaghi et al. exploited a novel imine exchange procedure to perturb the equilibrium^28^. Although such efforts towards polymorphic discovery are rare, we tried to exploit the reversible imine exchange possibility (Fig. 2-Bottom) for the present system, especially due to the presence of an aldehydic group (-CHO) in an almost orthogonal position to the molecular long axis. The experiments were performed by adding different amounts of amines in a given solvent (MeOH), where **Form I** was obtained earlier. The obtained solids were observed to differ with the incremental addition of benzylamine **(2)** to the methanol solution. Since competitive salt formation is possible *vs*. reversible imine formation, the crystallization outcome from a solution with a higher % of **2** was studied first. Crystallization from a solution with a higher % of added amines (>50%) yielded powder solids that matched with a previously reported **Bza**-**2** salt (JIFXIJ)^29^, indicating acid-amine salt formation was more prominent in the given concentration. However, a new phase was obtained at a lower % of added 2, when the % of added **2** in MeOH was varied between 8 and 12%, which neither matched with **Form I** nor **Bza**-**2** salt. The above results indicate a facile imine exchange may act as an inhibitor for the crystallization of **Form I**, leading to possibly a novel form. This was further monitored through a ^1^H NMR experiment, under similar conditions, which also indicated an exchange reaction (Supplementary Fig. S32)

This exchange process, however, became very weak at extreme dilution, and **Form-I** was crystallized. It must be noted here that the above observations can be specific to solvent and other experimental conditions. Similar experiments carried out with 10% benzylamine in other solvents (such as dichloromethane or acetonitrile) (Supplementary Fig. S14) resulted in the isolation of **Form I**. This type of difficulty may be expected, however, in the crystallization of metastable polymorphs^30^. To obtain structural insights into this new solid, we attempted to grow single crystals of the same in the next step. Table 1.

**Table 1:** Geometrical parameters associated with structures Bza-1 (Form I and Form II)

Structure	d_1_(Å)	τ (°)	d_2_(Å)	α(°)	Γ (°)
**Parameter**	**O**_**1**_**-N**_**1**_	**C**_**1**_**-O**_**1**_**-N**_**1**_**-C**_**2**_	**C**_**3**_**-C**_**5**_	**C**_**3**_**-C**_**5**_**-C**_**6**_	**C**_**3**_**-C**_**4**_**-C**_**5**_**-C**_**6**_
**Form I**	2.600 (2)	5.0 (1)	6.625 (2)	135.46 (9)	0.00 (3)
**Form II**	2.557 (2)	12.1 (2)	3.950 (4)	105.2 (2)	0.00 (8)/ 0

(O-N distance (*d*1) and torsional angle (*τ*) between carboxylate-pyridinium synthon, the distance between the two neighboring C=C bonds (*d*_2_), the angle (*α*) between and torsional (Γ) angle between the nearest C=C bond.

### Crystal packing analysis of Bza-1-Form II (Form II)

**Fig. 3:**
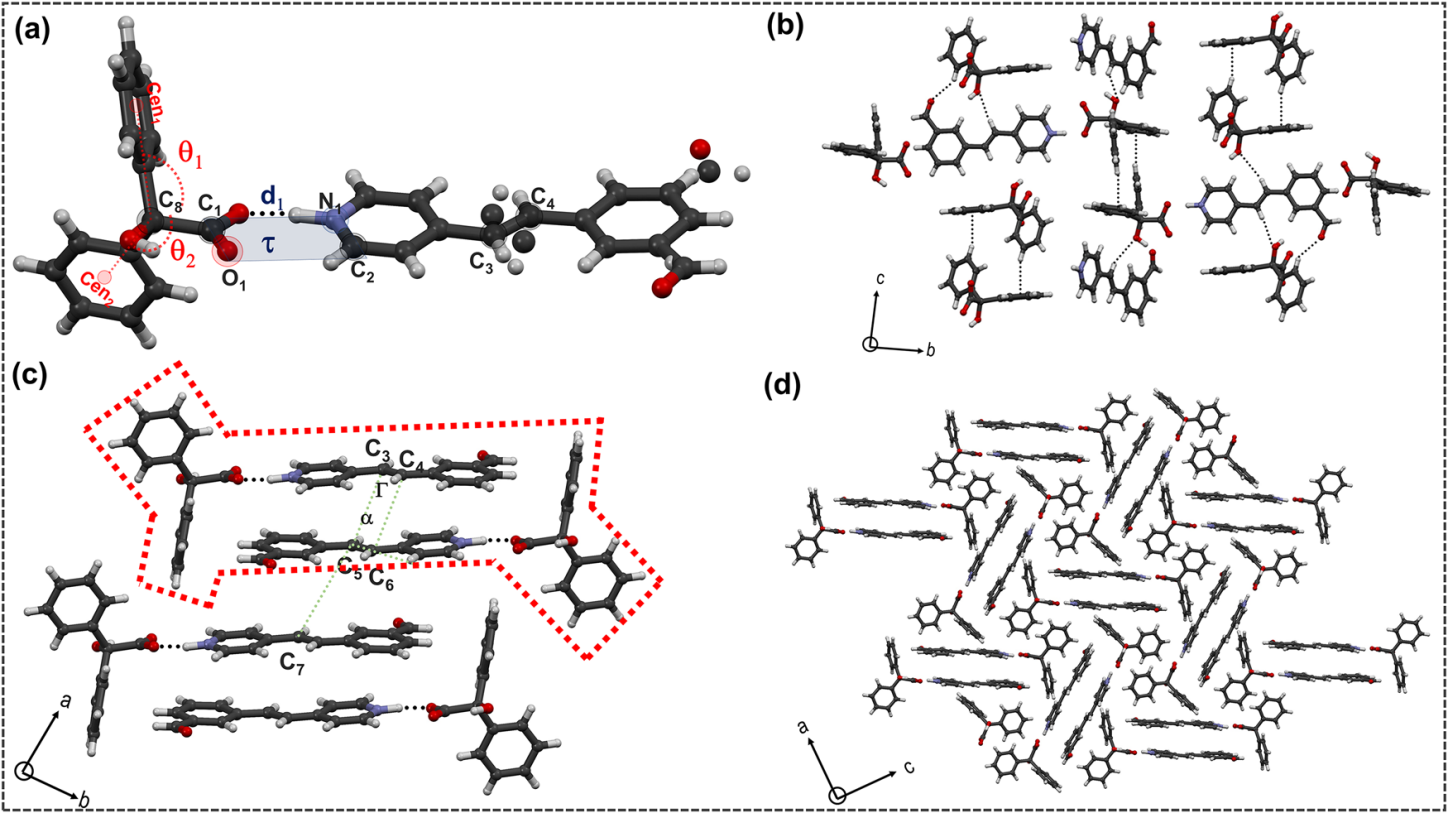
Packing features for Bza-1-Form II. **a** primary synthon, **b**, **c**, **d** packing diagrams of **Bza-1-Form II** along different crystallographic directions. LSAM 2 is marked with a red dotted line in (**c**).

The solution crystallization of **Bza** and **1** (1:1) from 10% of **2** in methanol resulted in colorless block-shaped crystals of **Bza** and **1**, in which molecules of **1** were packed in a *head-to-tail (h-t)* orientation (**Form II**) (Supplementary data 2). In this form, the primary synthon is formed again through a charge-assisted O^-^···H$-$N^+^ (1.55(3) Å) hydrogen bond. The torsional angle between the respective carboxylate-pyridinium synthon was found to be *τ* = 12.1(2)° (Fig. 3). It may be assumed that through the imine exchange, as described in the previous section, the access of -CHO was temporarily blocked during nucleation, leading to the typical LSAM-1 as observed in the earlier cases (Scheme 1). There are two interesting features in this structure. The olefinic carbons (C3, C4) in **Form II** are sustained through a positional disorder (with 93% and 7% occupancy in two positions). Such unequal occupancy in disordered atomic positions can be indicative of a dynamic equilibrium during nucleation rather than a pedal motion^31^, since **Form I**, under similar conditions, did not show any such disorder. The participation of an aldehydic-O in a weak C−H···O contact (2.424 Å) with one of the phenyl-H of **Bza** should also be noted. More detailed crystallographic experiments will be required, however, to completely rule out the possibility of a pedal motion in this case. The distance between the two olefinic groups lies around 3.950(4) Å, satisfying Schmidt’s topochemical criteria (<4.1 Å) to undergo photodimerization.

### Thermal analysis of the two forms

**Fig. 4:**
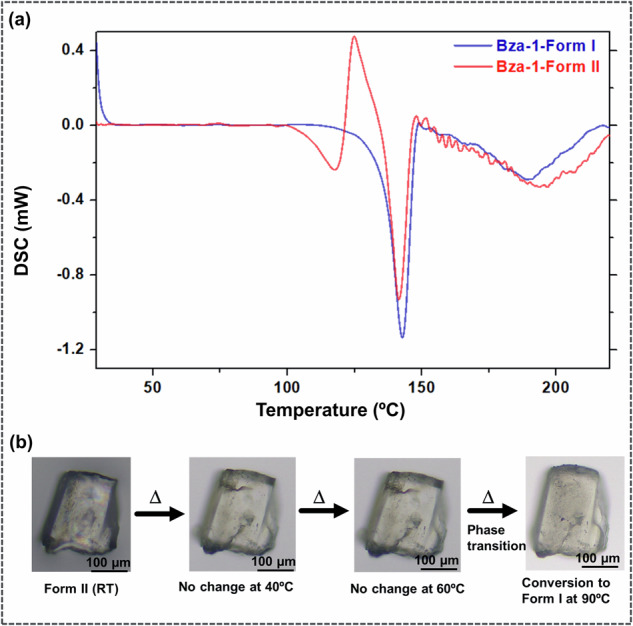
Thermal analysis of Form I and Form II. **a** DSC thermograms of **Form I** and **Form II** crystals. **b** Microscopic images capturing the conversion of **Form II** crystals to **Form I** upon heating.

The thermal characteristics of the obtained forms were investigated through Differential Scanning Calorimetry (DSC). DSC analysis of **Form I** showed no significant thermal event till its melting (T_m_ (Form I) = 143.08 °C, onset = 136.60 °C, Δ*H*_f_ = −51.65 J/g). The DSC thermogram of **Form II**, however, showed a small endotherm in a range between 96.5 °C to 124.8 °C, followed by melting at 141.7 °C (onset = 134.0 °C; Δ*H*_f_ = −51.64 J/g) (Supplementary Figs. S17, S18). This indicates a solid-state polymorphic transition of **Form II** to **Form I** at a temperature of 96.5 °C (Fig. 4).

To verify the phenomenon, a single crystal of **Form II** was heated in a hot stage microscope, and microscopic images were captured at different intervals between 40 °C and 100 °C. A cell check on the respective single crystals was carried out through a Single-crystal X-ray diffractometer. This revealed that a solid-state transition from **Form II** to **Form I** was observed at a temperature of ∼90 °C, which corroborates well with the DSC observations.

### Photochemical behavior of the polymorphic forms

**Fig. 5:**
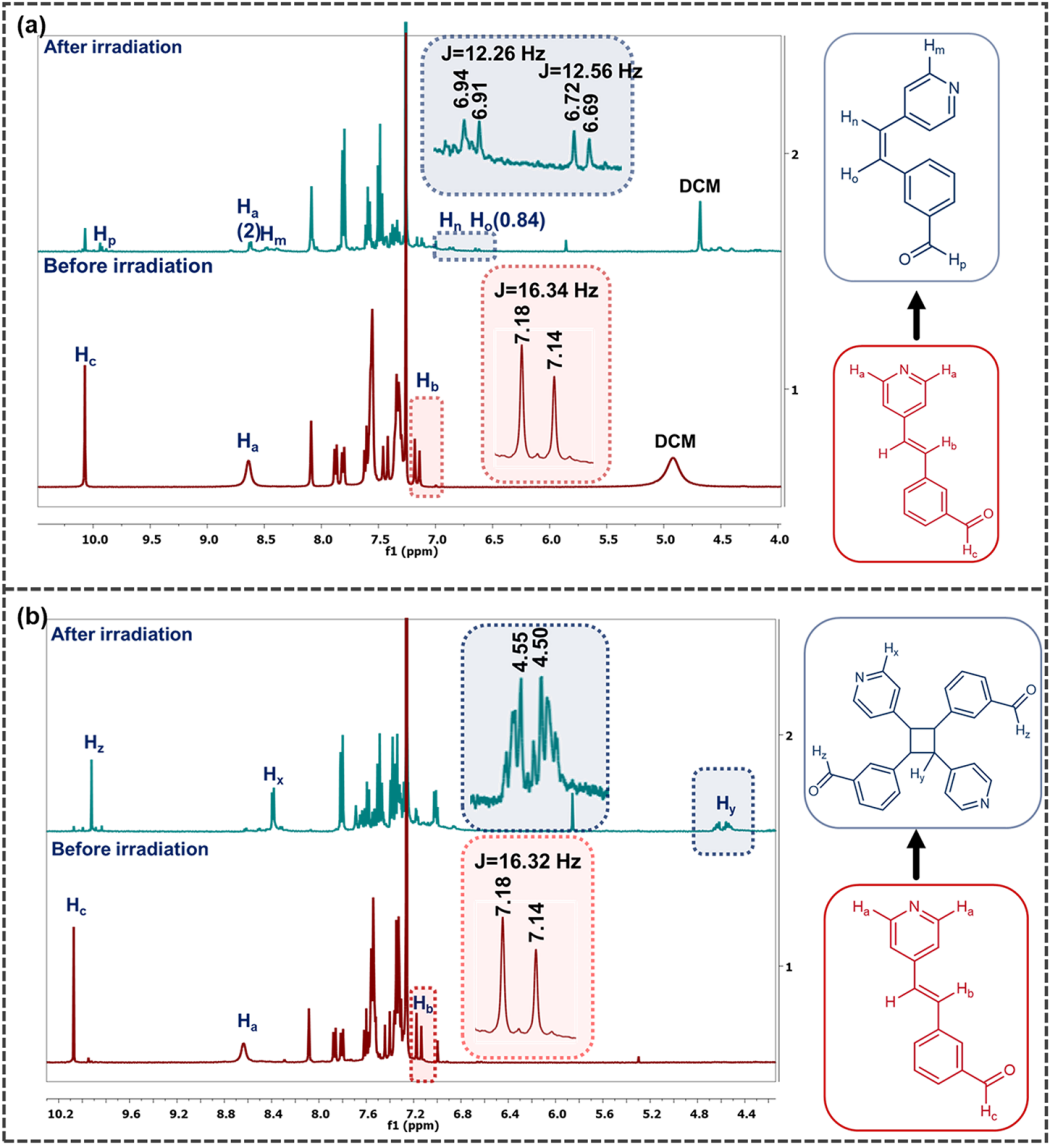
^1^H NMR analysis of the irradiated products. **a** **Form I**: The appearance of H_n_ and H_o_ protons in the irradiated sample indicates the formation of *cis* isomer. **b** **Form II**: The upfield shift of H_a_ proton to H_x_ and the appearance of H_y_ peak upon irradiation indicate the formation of an *h-t* photodimer.

A powder sample of **Bza-1-Form I** was irradiated under broadband UV radiation for 12 h. The analysis of ^1^H NMR and LC-MS spectra indicates the absence of a photo-dimer. Additional peaks, however, appeared at *δ* = 8.47–8.49 ppm (*H*_m_), along with the pyridine hydrogen at *δ* = 8.62–8.63 ppm (H_a_). Another set of peaks that appeared at *δ* = 6.91–6.94 ppm (*H*_n_) and *δ* = 6.69–6.72 ppm (*H*_o_) (Fig. 5a), with the coupling constants (*J*) of 12.26 Hz and 12.56 Hz, respectively, suggest the formation of the *cis* isomer of the **1**. Integration of *H*_n_ and *H*_o_ with respect to the H_a_ proton (2H, doublet) suggests a partial conversion of the *trans*-isomer to the *cis*-isomer upon 12 h of photo-irradiation (Supplementary Note 4).

Powder samples of **Bza-1-Form II** were also irradiated under broadband UV radiation for 12 h. The **Bza-1-Form II** crystals formed respective *h-t* photodimers in quantitative yield. In the ^1^H NMR spectrum, the appearance of new peaks at *δ* = 8.31–8.33 ppm (H_x_) and *δ* = 4.50-4.55 ppm (H_y_) indicates [2 + 2] *h-t* photo dimer formation (Fig. 5b). Comparable integration of H_x_ and H_y_ protons, along with the disappearance of the H_a_ protons, suggests complete conversion of **1** to an *h-t* photoproduct.

### Photomechanical responses of the two polymorphic forms

**Fig. 6:**
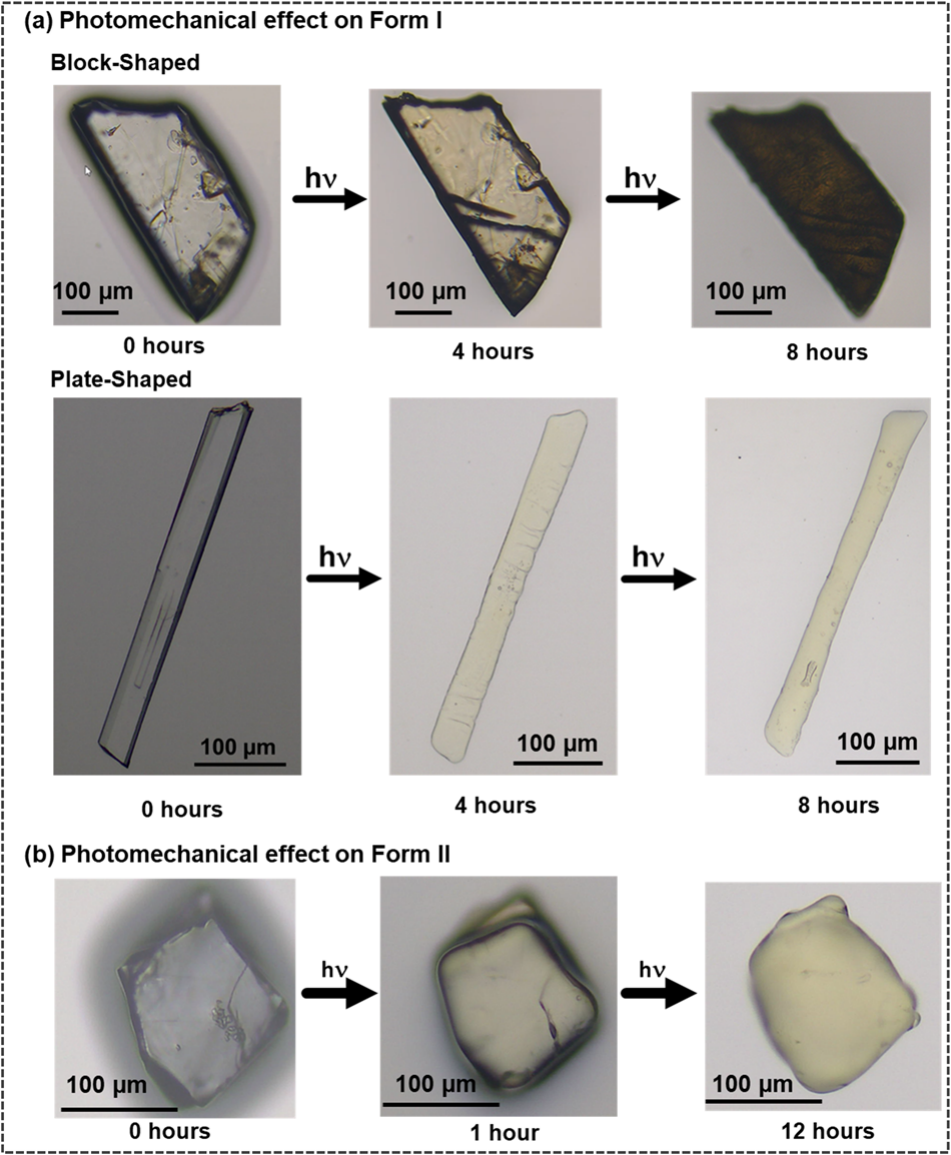
Optical microscopic images of the irradiated crystals. **a** **Form I** block-shaped crystals obtained from methanol showed photoinduced cracking during irradiation under broadband UV light. **Form I** plate-shaped crystals obtained from nitromethane showed photoinduced melting upon irradiation under broadband UV. **b** **Form II** crystals showed photoinduced solid-to-liquid transition during irradiation under broadband UV light.

The single crystals of both forms were irradiated under UV light to investigate their respective photomechanical properties. As mentioned, **Form I** was crystallized in two different morphologies: block-shaped crystals were obtained repeatedly from many solvents (Supplementary Methods (S1)), and plate-shaped crystals were formed from nitromethane. When a block-shaped single crystal of **Bza-1-Form I**, obtained from methanol, was exposed to broadband UV radiation, cracks were observed on the surface of the crystals after four hours of irradiation. With the increase in irradiation time, the color of the crystal was gradually changed to black, retaining the overall morphology (Fig. 6a). When the plate-shaped crystals of **Bza-1-Form I** were irradiated, a solid-to-liquid transformation was observed after ∼4 h of irradiation that was completed in ∼8 h. The LC-MS spectra of the melt suggested no dimer formation, which corroborates well with the ^1^H NMR analysis in the preceding section (Supplementary Note 5).

Since the molecules of **1** are situated diagonally with respect to the major (001) plane of **Form I**, the generation of diagonal cracks may be explained due to the cis-trans isomerization on the (001) surface. From the face indexing diagrams (Fig. 1f), it can also be assumed that partial *trans-cis* transformation of the molecules of **1** will disrupt the C−H···O interactions between neighboring molecules. Since photo-induced changes are often propagated by the crystal surface^32^, the block-shaped crystals turn black, while plate-shaped crystals undergo a solid-to-liquid transition upon prolonged irradiation. This is the first time we observed a solid-to-liquid transition in this class of compounds due to *trans*-*cis* isomerization^33^.

While exposed to broadband UV radiation, **Form II** crystals also underwent solid-to-liquid transformation. Optical microscopic images suggest that the transformation may have started after 1 h and was completed in 12 h. As mentioned in a previous section, **Form II** sustains through a herringbone packing and is expected to undergo a solid-to-liquid transition since such packing dictates the absence of a slip plane. This observation is in accordance with the previous results obtained in this class of compounds^18^. The obtained liquid was further checked through LC-MS, and a peak at m/z = 419 suggested the formation of a [2 + 2] photodimer (Supplementary Note-5, Supplementary Fig. S34).

### Photophysical behavior of the polymorphic forms

**Fig. 7:**
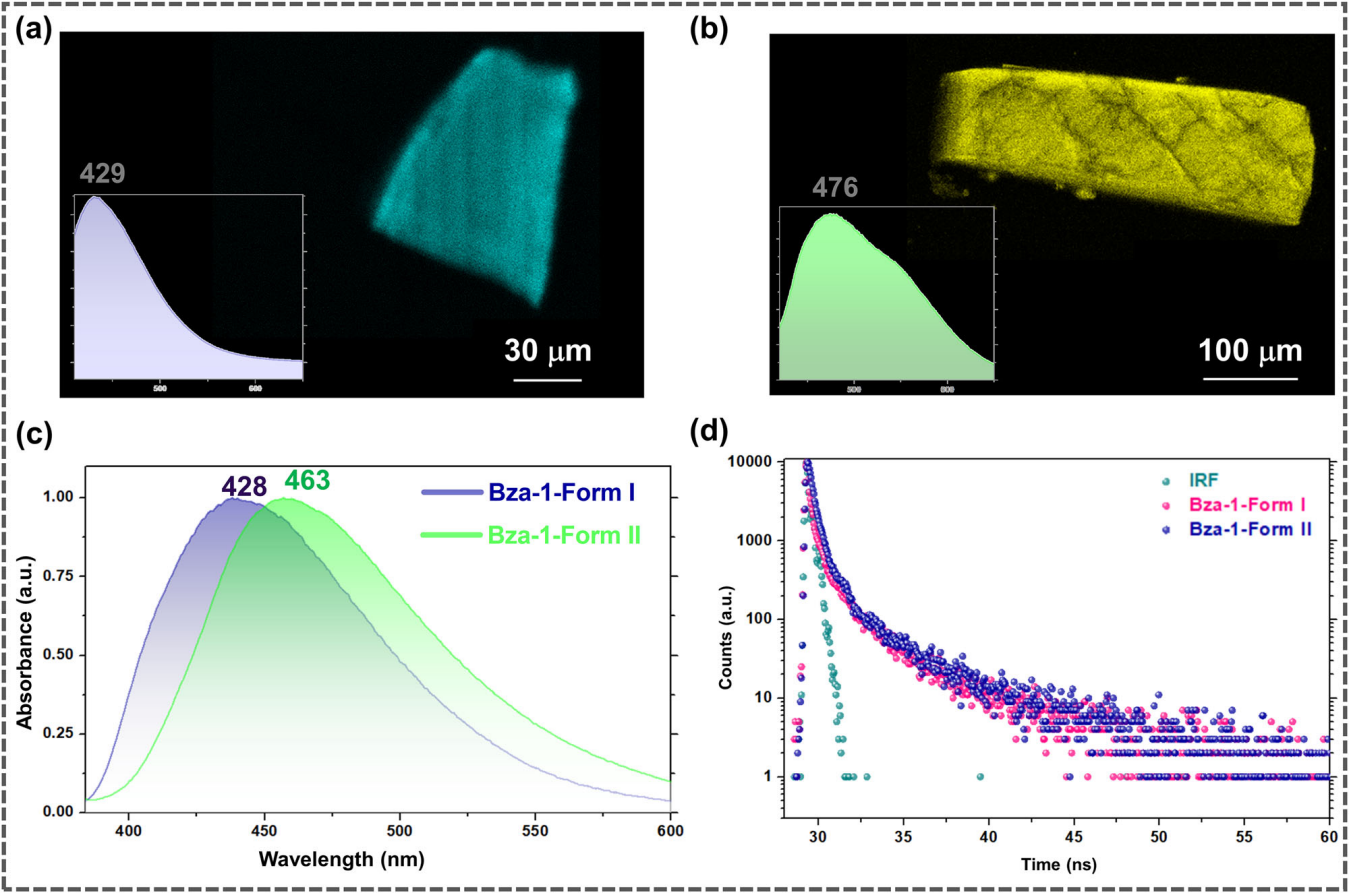
Photophysical properties. Confocal microscopic images of **a** **Form I** and **b** **Form II** (the pseudo color was chosen based on the xy vs. λ scan spectra. **c** Photoluminescence spectra of **Form I** and **Form II**. **d** Decay plot of **Form I** and **Form II**.

Solid-state UV-vis absorption and fluorescence spectra were analyzed to delineate the photo-physical properties of the two forms. The absorbance maxima of **Form II** (383 nm) were found to be red-shifted compared to **Form I** (365 nm), indicating increased intermolecular charge transfer (CT) interactions between the coformers in **Form II** compared to **Form I** (Supplementary Note-7). In photoluminescence spectra, **Form I** and **Form II** showed emission maxima at 428 and 463 nm, respectively (Fig. 7c). These results agree well with confocal microscopic images, in which the xy vs. λ scans showed a maximum emission at 429 and 476 nm, respectively, for **Form I** and **Form II** single crystals (Fig. 7a, b). Time-correlated luminescence spectra for the two forms suggest a longer average lifetime of **Form II** (0.1727 ns) compared to **Form I** (0.0713 ns), again indicating greater CT interactions in **Form II**.

Further, DFT-based frontier molecular orbital calculation was performed on both forms using coordinates from respective structures. The highest occupied molecular orbitals (HOMO) were constituted on the **Bza** molecule, with an energy level of -6.32 eV for **Form I** and −6.13 eV for **Form II**. The lowest unoccupied molecular orbital (LUMO) was concentrated on **1**, with an energy level of −3.76 eV for **Form I** and −3.79 eV for **Form II** (Supplementary Note-6). The results indicate that CT interactions may occur between HOMO of **Bza** to LUMO of **1**. These results agree with the observations from UV–vis spectral profiles.

## Conclusion

Several conclusions can be derived from the current study, which are of future interest. (1) utilizing strong acceptors in almost orthogonal directions influences large synthon formation, due to a donor-acceptor imbalance. As a result, the robust LSAM (LSAM-1) that was observed in previous studies is somewhat inhibited and a different LSAM was formed (LSAM-2) in the more stable polymorph (**Form I**). This idea to manipulate LSAM formation is itself novel. This concept can be profitably extended to other systems in the future for directed polymorphism studies. (2) The two obtained solids, derived through differing LSAMs, showed drastically different photochemical properties. While irradiation of **Form I** does not lead to photo dimers, instead undergoes partial *cis-trans* isomerization, photo-irradiation of **Form** **II** leads to a photo dimer formation. This is the first time, to our knowledge that two polymorphic forms of a multicomponent solid showed such distinct differences in photochemical behavior. (3) The differences in large synthons also manifest in photomechanical behavior. **Form I** sustains different photomechanical responses depending on crystal morphology, while **Form II** undergoes a solid-to-liquid transition. The photo-induced melting in **Form I** plate-shaped crystals seems to arise from a *trans-cis* isomerization, as per the packing analysis. While photo-induced solid to liquid transformation itself is a rare occurrence in multicomponent solids, with only a few examples at hand^18,34^, examples of such phenomena arising from different origins in two polymorphs in a multi-component solid are hitherto unknown to our knowledge. In addition to the above points, utilization of imine exchange reaction to facilitate the formation of **Form II** is also interesting. Although such protocols were previously used in COF chemistry^28^, applications of it in polymorph screening to isolate a novel polymorph of a multicomponent functional solid are rare.

Overall, this study highlights that tuning LSAMs can be a feasible way to engineer multiple properties at the same time. This aspect touches upon a much-sought area of crystal engineering where retrosynthesis may start from targeted properties rather than a structure. Often termed third-generation crystal engineering^35^, this field extends the scope of crystal engineering to a broader perspective and integrates it with materials science.

## Methods

### (I) Synthesis of (E)-3-(2-(pyridin-4-yl) vinyl) benzaldehyde (1)

The compound was prepared using a reported procedure^36^: 10 mmol of 4-methylpyridine (0.94 g) and 10 mmol of isophthaldehyde (1.34 g) were weighed in a dry round-bottom flask. 2 mL propionic anhydride was added to the mixture, and the reaction mixture was refluxed at 140 °C for 48 h. The dark brown color crude product obtained was dissolved in DCM, and a saturated sodium bicarbonate solution was added to neutralize the excess acid. Column chromatography was used to purify the compound using 20% EtOAc/hexane solution. ^1^H NMR studies of the purified compound are provided in the Supplementary Methods (S1).

### (II) Crystallization experiments

#### (a) Preparation of Bza-1-Form I

Form I was obtained through several procedures, including solution crystallization, melt crystallization, and liquid-assisted grinding (LAG). The details these protocols are provided in Supporting Information (Supplementary Methods (S1)).

#### (b) Preparation of Bza-1-Form II

A 0.05 M benzylamine solution in methanol (2000 µL) was prepared. In separate glass vials, 0.1 mmol of benzilic acid (**Bza**) (22.3 mg) and 0.1 mmol of (E)-3-(2-(pyridine-4-yl) vinyl) benzaldehyde (**1**) (20.9 mg) were accurately weighed. Solution crystallization was performed by dissolving the **Bza** and 1 mixture with varying amounts of 0.05 M benzylamine solution. For instance, to perform crystallization from 2% benzylamine solution, a 0.1 mmol mixture of Bza and 1 was dissolved in 40 µL of the prepared 0.05 M benzylamine solution and subjected to solvent evaporation. The volume of 0.05 M benzylamine solutions used in other cases are 120 µL (6%), 160 µL (8%), 200 µL (10%), 240 µL (12%), 400 µL (20%), and 1400 µL (70%) respectively.

### (III) Crystallographic studies

#### (a) Single crystal X-ray diffraction

Single crystal data were collected for both samples using a Rigaku Oxford diffractometer, using a Cu-Kα = 1.54184 Å radiation, at 300 K. The data was processed using Crysalis Pro^37^ software. Structures were solved using SHELXT (intrinsic phasing) embedded in OLEX2^38^ software. Non-hydrogen atoms were refined anisotropically using the SHELXL^39^ package embedded in the same software. Hydrogen atoms were identified through the riding model, except the labile hydrogens. Labile hydrogens were identified through the Fourier map. The face indexing of both block and plate-shaped crystals was performed in the same diffractometer at 300 K. In this case, the major faces were identified as (001/00-1) along with several minor faces. Additional crystallographic information (crystallographic table and hydrogen bonding table) is provided in Supplementary Note 1.

#### (b) Powder X-ray diffraction (PXRD)

The PXRD data was collected using a Rigaku Ultima IV diffractometer, within a range between 5-40°, with a scan speed of 2°/min and a step size of 0.02. The simulated pattern was generated using the collected structure through mercury, and stacked patterns were plotted using X’Pert high score plus software.

### (IV) Thermal analysis

The differential scanning calorimetry (DSC) data for the PXRD-matched sample were collected using a Shimadzu instrument. Heating was done in a sealed aluminum pan ranging between 30 °C to 250 °C with a ramp rate of 5 °C/min. The DSC instrument was calibrated using indium before the sample ran.

### (V) Photochemical studies

The powder samples of Form I and Form II were transferred to a glass slide and irradiated with broadband UV light for 12 h. After 12 h, the sample was used for ^1^H NMR and LC-MS. ^1^H NMR data were collected using a Bruker (400 MHz) instrument by dissolving the native and irradiated samples in CDCl_3._ The LC-MS data were collected by dissolving the samples in methanol using a Shimadzu 8000 instrument.

### (VI) Photophysical studies

Absorbance spectra of the PXRD-matched solid samples were collected using a JASCO spectrophotometer within a range between 200 and 600 nm. Depending on the absorbance maxima, the solid-state luminescence spectra were recorded using a JASCO Flurolog instrument with a slit width of 2 nm. TCSPC of the samples was collected using a Horiba Delta flux-1, depending upon the excitation and emission spectra of the samples, and the decay curves were fitted using Ez-Time analysis software. The confocal images of the respective single crystals were collected using a Leica confocal microscope, with an excitation laser of 405 nm with 10% power. An xy vs. λ scan was performed to determine the color of the crystal exactly. The xy vs. λ scan was performed with a range between 420 and 650 nm, with a detection step size of 5 nm. Images were processed using LAS-X software.

### (VII) Microscopic analysis

The images of the well-grown single crystals, before and after irradiation, were collected using an Olympus microscope.

## Supplementary information

Supporting Information

Description of Additional Supplementary Files

Supplementary Data 1

Supplementary Data 2

Supplementary Data 3

## Data Availability

The X-ray crystallographic coordinates for structures (Bza. 1Form I and Form II), as reported in this article, have been deposited at the Cambridge Crystallographic Data Centre (CCDC), under deposition numbers CCDC 2419702, 2419704. These data can be obtained free of charge from The Cambridge Crystallographic Data Centre via www.ccdc.cam.ac.uk/data_request/cif. Details of syntheses, cocrystallization, crystallographic tables, additional NMR spectral analysis, PXRD diffractograms, and molecular modeling are provided in the Supplementary Material. All relevant data are available from the authors upon request. Crystallographic information files for Form I and II are provided as supplementary data-1 and 2, respectively. NMR spectra are provided as supplementary data-3.
