# Supplementary material for: Regulating photoreactivity in a polymorphic bi-component solid through large synthons
Source: Commun Chem. 2025 Apr 30;8:130. doi: 10.1038/s42004-025-01527-w (PMC12043805; doi:10.1038/s42004-025-01527-w)
Supplement: Supplementary file 1 — Supporting Information [file 42004_2025_1527_MOESM1_ESM.pdf]

Regulating photoreactivity in a polymorphic bi-component solid through large synthons

Mollah Rohan Ahsan, Dr. Arijit Mukherjee*
Department: Department of Chemistry,
Institution: Birla Institute of Technology and Science, Pilani, Hyderabad Campus
Address 1: Jawahar Nagar, Kapra Mandal, Medchal District, Telangana 500078, India
E-mail: arijit.mukherjee@hyderabad.bits-pilani.ac.in

Table of contents:

Sl. No.	Title	Page No.
S1.	Syntheses and crystallization	S2-S5
S2.	Additional Crystallographic information	S5-S7
S3.	Additional PXRD characterization	S7-S14
S4.	Additional DSC analysis	S15
S5.	Additional NMR Spectra	S16-S25
S6.	LC-MS spectra	S26-S27
S7.	Computational Calculations	S27-S28
S8.	Additional Photophysical characterization	S28-S29
S9.	Supplementary References	S29-S30

S1. Syntheses and crystallization:

Supplementary Methods

The compounds used for the study are benzilic acid (**Bza**), (E)-3-(2-(pyridin-4-yl) vinyl) benzaldehyde (**1**), and benzylamine. The benzylamine is purchased from SRL chemicals, and benzilic acid and (E)-3-(2-(pyridin-4-yl) vinyl) benzaldehyde was prepared using a reported method. The detailed synthetic method is described below.

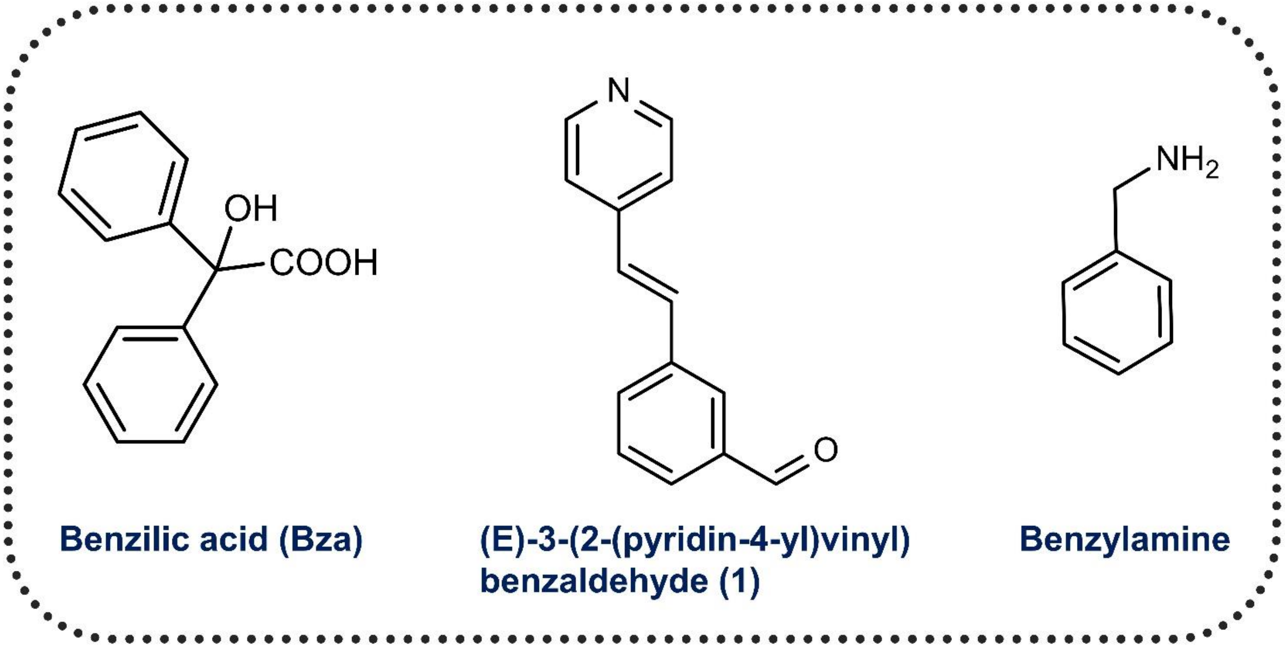

Figure S1: Compounds used for the study.

Synthesis of the coformer:

Synthesis of (E)-3-(2-(pyridin-4-yl) vinyl) benzaldehyde (1): The compound was prepared using a reported procedure: 10 mmol of 4-methylpyridine (0.94 g) and 10 mmol of Isophthalaldehyde (1.34 g) were weighed in a dry round-bottom flask. 2 mL propionic anhydrite is added to the mixture, and the reaction mixture is refluxed at 140 ºC for 48 hours. The dark brown crude product obtained was dissolved in DCM, and a saturated sodium bicarbonate solution was added to neutralize the excess acid that had formed. The final product was purified using 20% EtOAc/hexane solution through column chromatography.

^{1}H NMR spectrum of purified synthesized compound: All the respective proton peaks are assigned below.

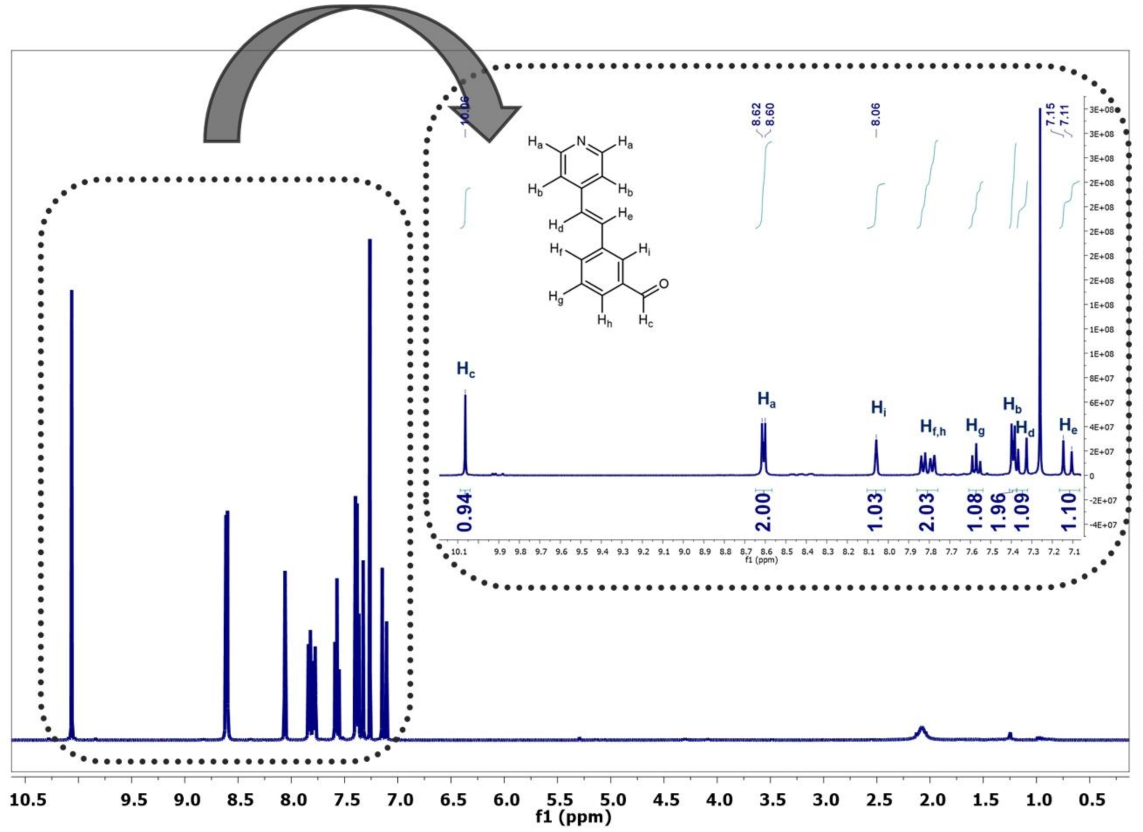

Figure S2: ^{1}H NMR spectra of purified **1.**

^{13}C NMR of the purified synthesized compound:

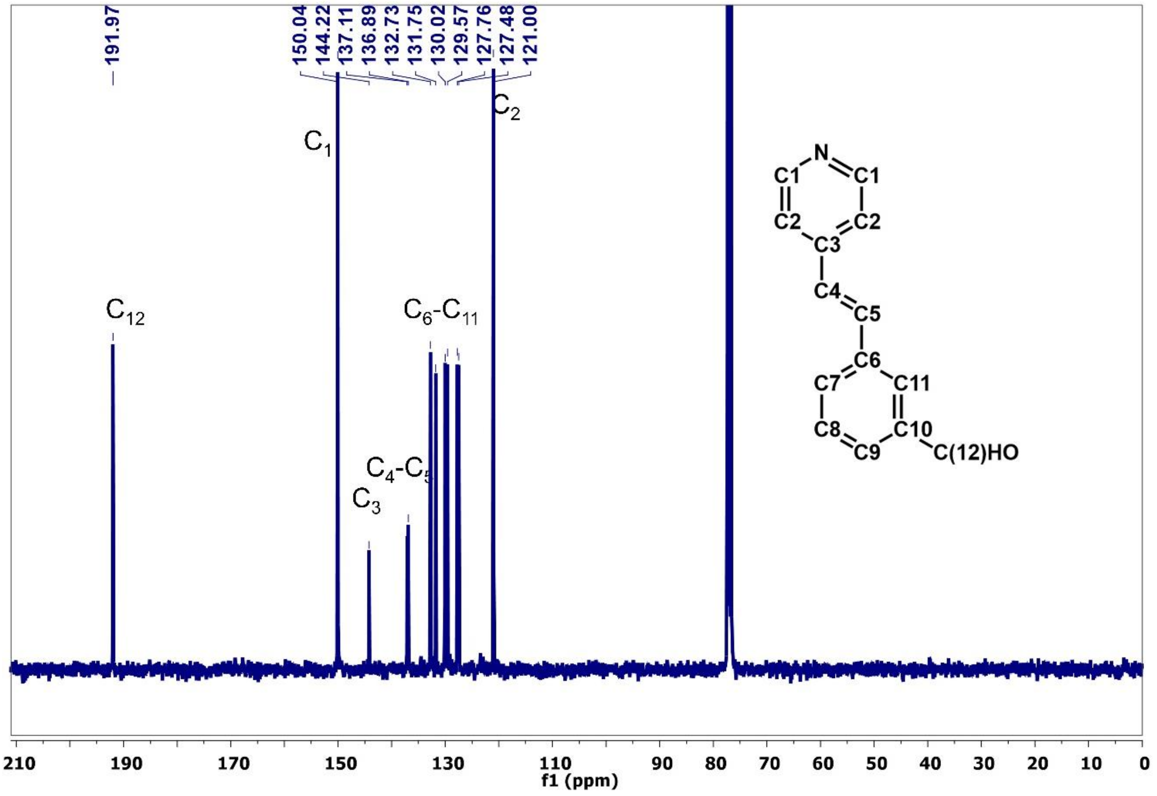

Figure S3: ^{13}C NMR spectra of purified **1**.

Crystallization:

Crystallization of the native 1:

The column-purified product was tried to crystallize through melt crystallization, solution crystallization, and LAG, followed by crystallization from different solvents like methanol, ethanol, acetonitrile (ACN), nitromethane, 1,4-dioxane, Dimethyl sulfoxide (DMSO), toluene, chloroform, and N,N′-dimethyl formamide (DMF). However, in all these cases, we were unable to produce single crystals for single-crystal X-ray diffraction.

Preparation of Bza-1-Form I:

Form I crystals were obtained through liquid-assisted grinding (LAG) followed by evaporation, through solution crystallization, and through melt crystallization. In the case of LAG, 0.1 mmol of **Bza** (22.7 mg) and 0.1 mmol of (E)-3-(2-(pyridine-4-yl) vinyl) benzaldehyde **(1)** (20.9 mg) were weighed in a mortar and ground with 3-4 drops of ACN. The ground mixture was dissolved in three parts and evaporated from 1. ACN (1 mL), 2. Ethanol (1 mL), 3. DCM (2mL). The single crystals obtained after 2 days from ACN and ethanol and after 1 day from DCM were used for single crystal analysis. Apart from that, melt crystallization, liquid-assisted grinding, and solution crystallization were performed to screen polymorphs.

For solution crystallization 0.05 mmol of **Bza** (11.3 mg) and 0.05 mol of (E)-3-(2-(pyridine-4-yl) vinyl) benzaldehyde (10.5 mg) was weighed and were dissolved in different solvents i.e., methanol, ethanol, isopropanol, ACN, nitromethane, toluene, 1,4-dioxane, DMF, DMSO, acetic acid, for solvent evaporation. Unit cell checks through single crystal diffraction indicated formation of Form I crystals.

The bulk phase purity of the crystals obtained through solution crystallization was checked for ACN, methanol, nitromethane and DCM, which showed the formation of Form I in the bulk phase. Melt crystallization and liquid-assisted grinding from ethanol, ACN, DCM, and 1,4-dioxane also produced Form I crystals in the bulk phase. Noticeably, the single crystals obtained from methanol, ACN, dioxane, toluene, DMSO, and DMF had a block morphology whereas the single crystals produced from nitromethane showed a plate morphology.

Preparation of Bza-1-Form-II:

A 0.05M, 2000 µl benzylamine solution in methanol (by dissolving 10 µL (0.1 mmol) benzylamine in a 2000 µL in methanol) was prepared. In separate glass vials, 0.1 mmol of benzilic acid (22.3 mg) and 0.1 mmol of (E)-3-(2-(pyridine-4-yl) vinyl) benzaldehyde (20.9 mg) were accurately weighed. Solution crystallization was performed by dissolving the **Bza** and 1 mixture with varying amounts of 0.05M benzylamine solution. In the case of 8% (160 µL benzylamine solution), 10% (200 µL benzylamine solution), and 12% (240 µL benzylamine solution), the diffractograms were different compared to Form I and Bza-benzylamine salt. The single crystals were obtained from the same solvent systems in our hand and used for single-crystal X-ray analysis.

S2. Additional Crystallographic information

Supplementary Note 1

Crystallographic Table:

**Compound**	**Bza-1-Form I**	**Bza-1-Form II**
Empirical formula	$C_{28} H_{23} N O_4$	$C_{28} H_{23} N O_4$
CCDC NO.	2419702	2419704
Formula Weight	437.47	437.47
Crystal System	$P\bar{1}$	$P2_1/c$
Space Group	*Triclinic*	*Monoclinic*
a (Å)	6.6250 (4)	8.8395 (3)
b (Å)	11.1098 (7)	17.8941 (6)
c (Å)	15.9599 (10)	14.6917 (8)
α (˚)	85.815 (5)	90
β (˚)	78.156 (5)	90.398 (4)

***γ* (°)**	77.597 (5)	90
***V* (Å3)**	1122.33 (12)	2323.80 (17)
***$ρ_{calc}$* (g/cm3)**	1.294	1.250
***F* (000)**	460.0	920.0
***μ*. (mm-1)**	0.699	0.675
***T* (K)**	300	298
***λ* (Å)**	1.54184	1.54184
Total Reflections.	4712	5009
Unique Reflection	4081	3515
Completeness (%)	99.6	100
***R_{int}* (%)**	2.45	4.04
R_1 (F^2) (%)	4.37	5.66
wR_2(F^2) (%)	12.95	18.96
GoF	1.093	1.072
***$Θ_{max}$* (°)**	79.687	80.491

Hydrogen Bonding Table:

structure	interaction	D-H (Å)	H⋯A (Å)	D⋯A (Å)	<(DHA) (°)
**Bza-1-Form I**	N5-H5⋯O1<br>O3-H3⋯O1	1.07(2)<br>0.92(3)	1.54(2)<br>1.82(3)	2.6003(14)<br>2.5549(14)	175.7(19)<br>135(2)
**Bza-1-Form II**	N4-H4⋯O1<br>O3-H3⋯O2	1.01(3)<br>0.82	1.55(3)<br>2.05	2.5573(19)<br>2.571(2)	171(3)<br>121.1

ORTEP diagrams: ORTEP diagrams were plotted with 50% probability.

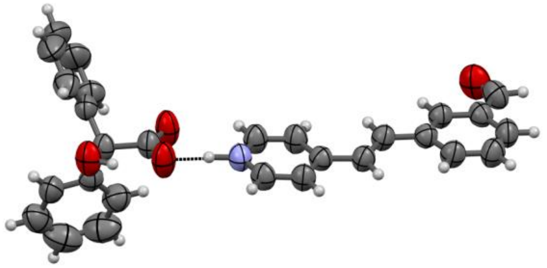	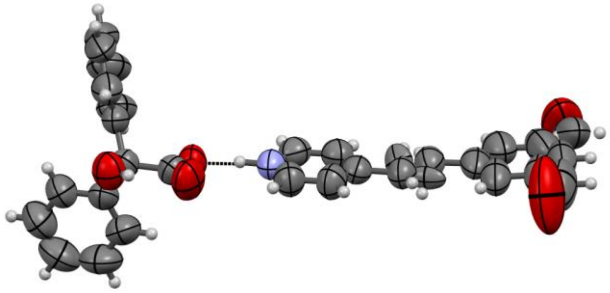
**Bza-1-Form-I**	**Bza-1-Form-II**

S3. Additional PXRD characterization

Supplementary Note 2

Bza-1-Form I bulk phase characterization

Liquid Assisted Grinding (LAG):

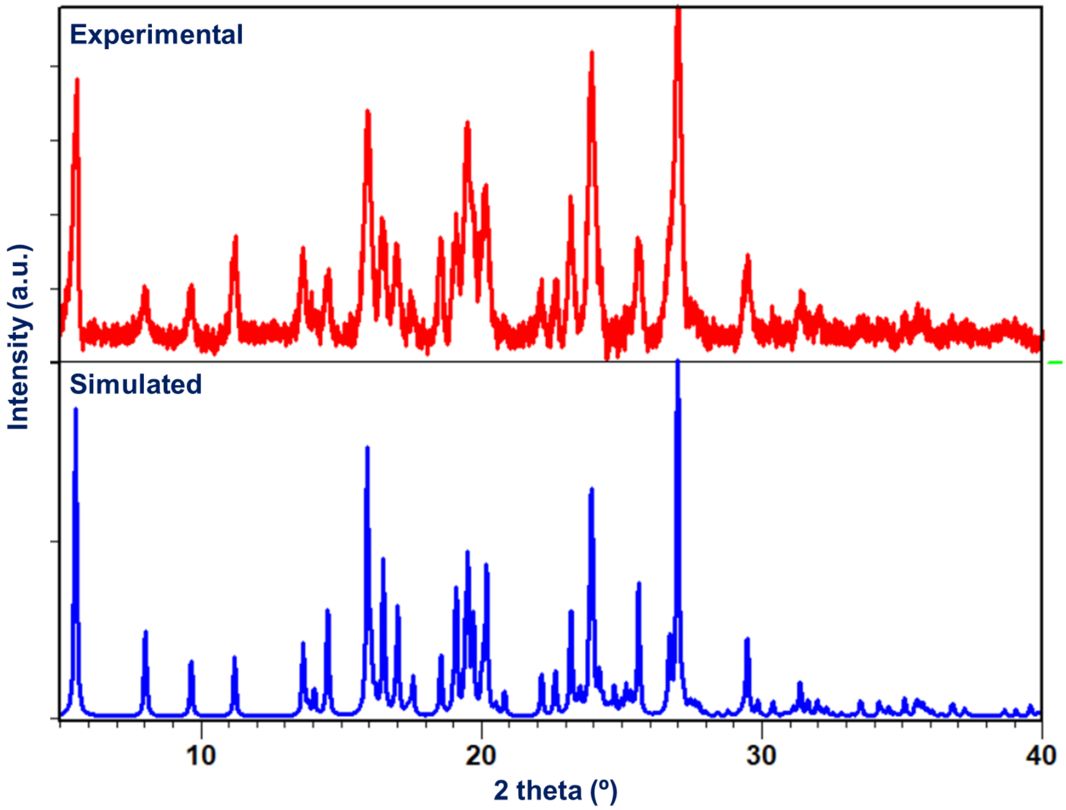

Figure S4: PXRD data of **Bza-1-Form I** obtained through LAG using ACN.

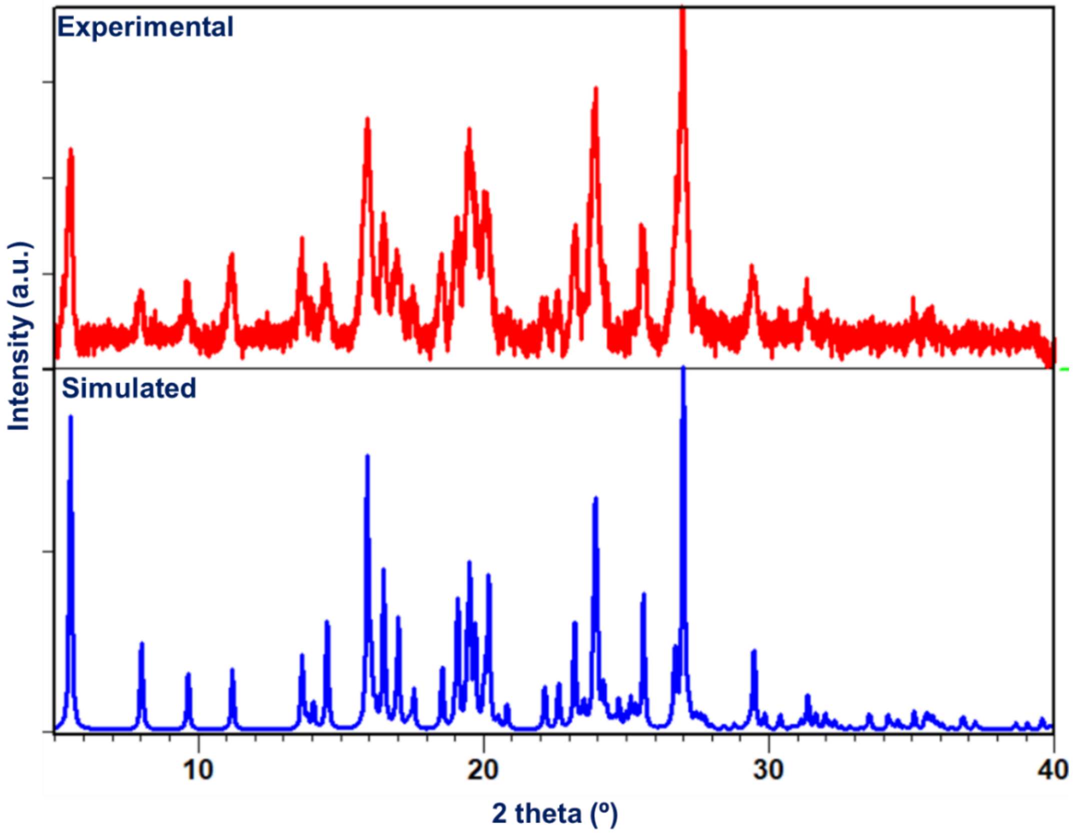

Figure S5: PXRD data of **Bza-1-Form I** obtained through LAG using DCM.

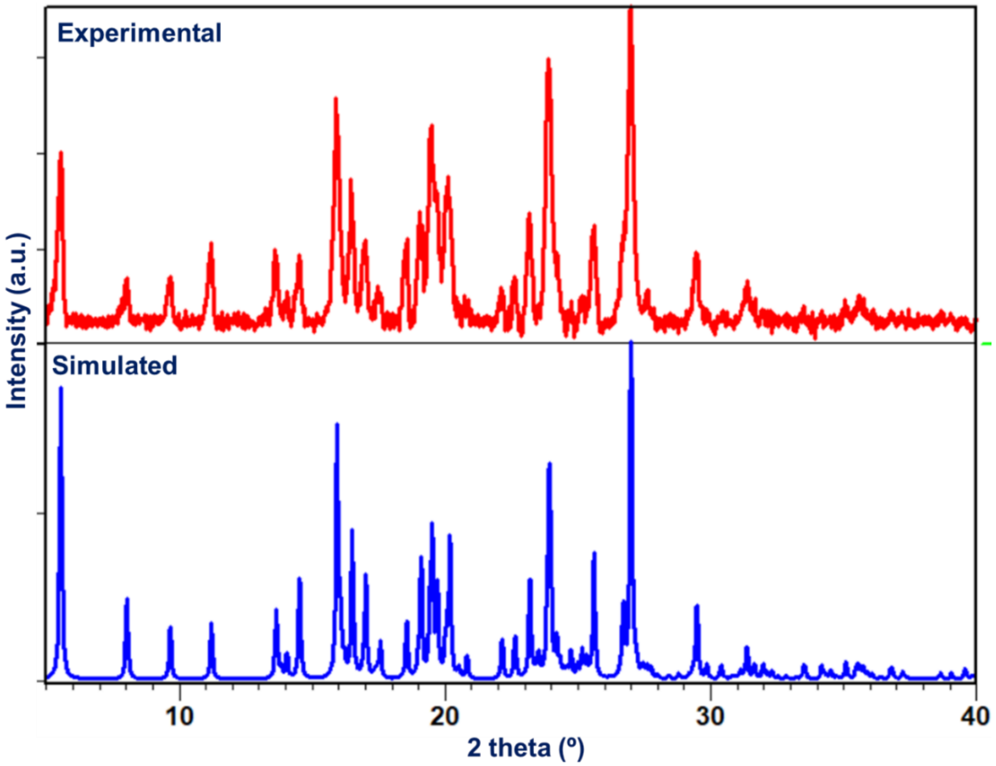

Figure S6: PXRD data of **Bza-1-Form I** obtained through LAG using ethanol.

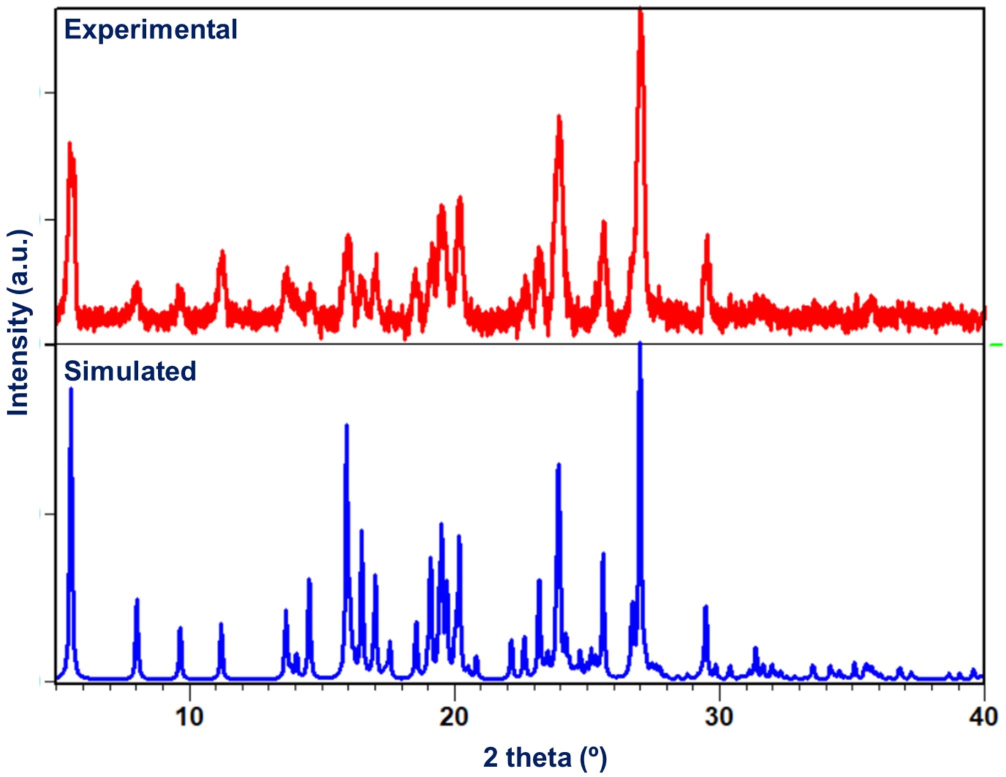

Figure S7: PXRD data of **Bza-1-Form** I obtained through LAG using 1,4-dioxane.

Melt crystallization

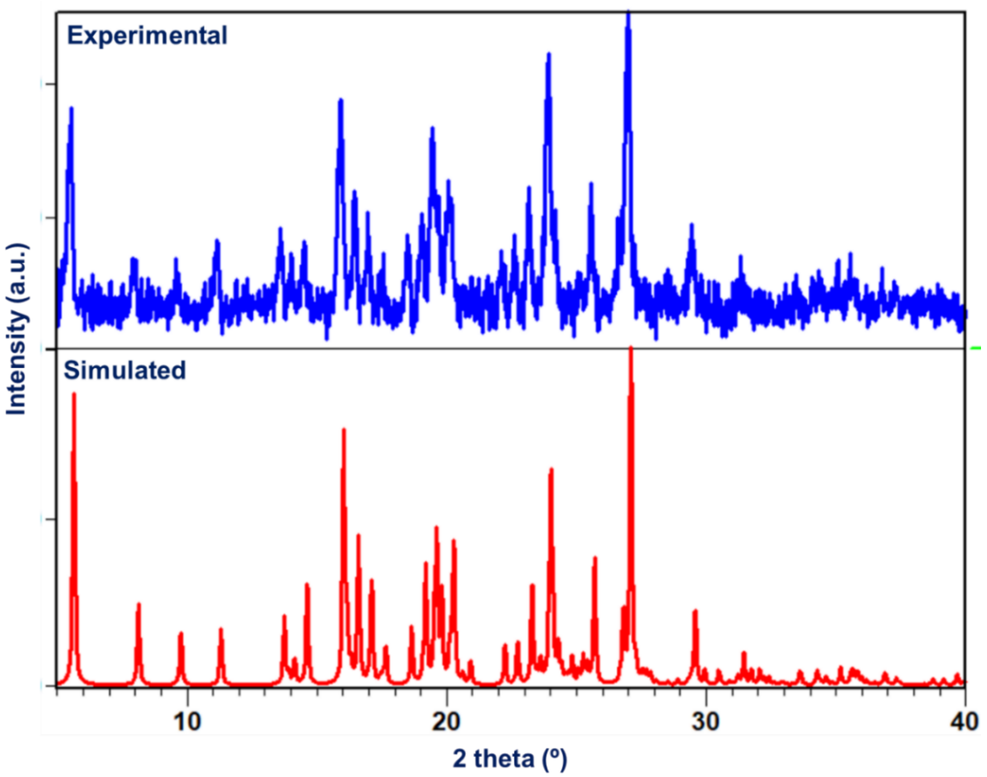

Figure S8: PXRD data of **Bza-1-Form I** obtained through melt crystallization.

Solution crystallization

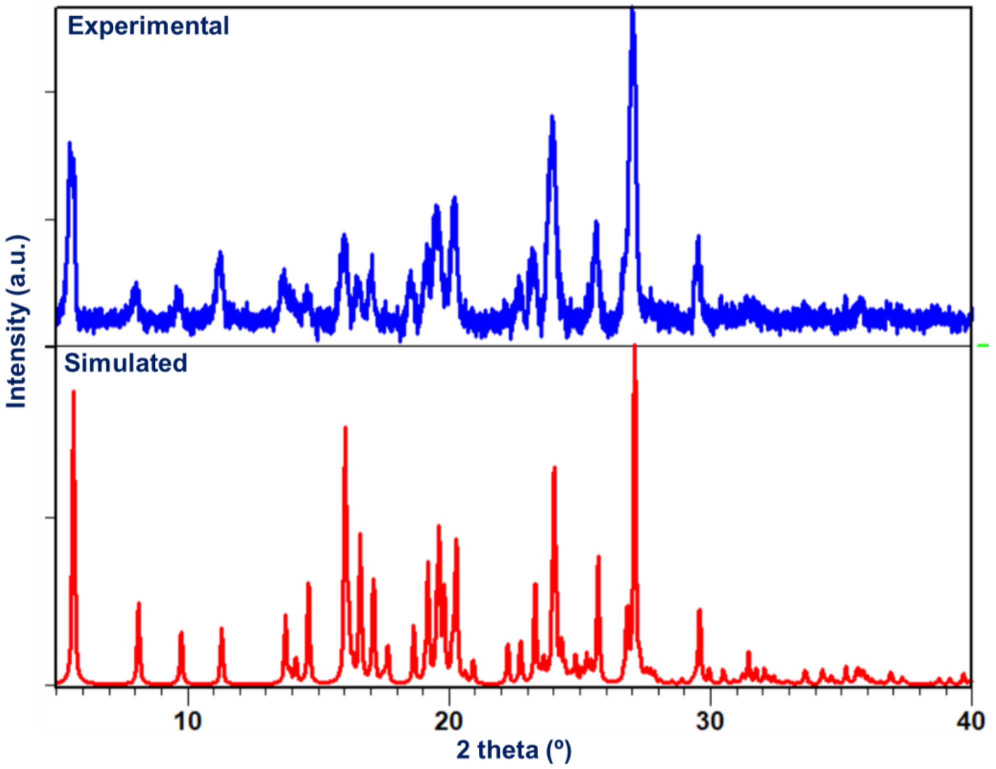

Figure S9: PXRD data of **Bza-1-Form I** obtained through solution crystallization from DCM.

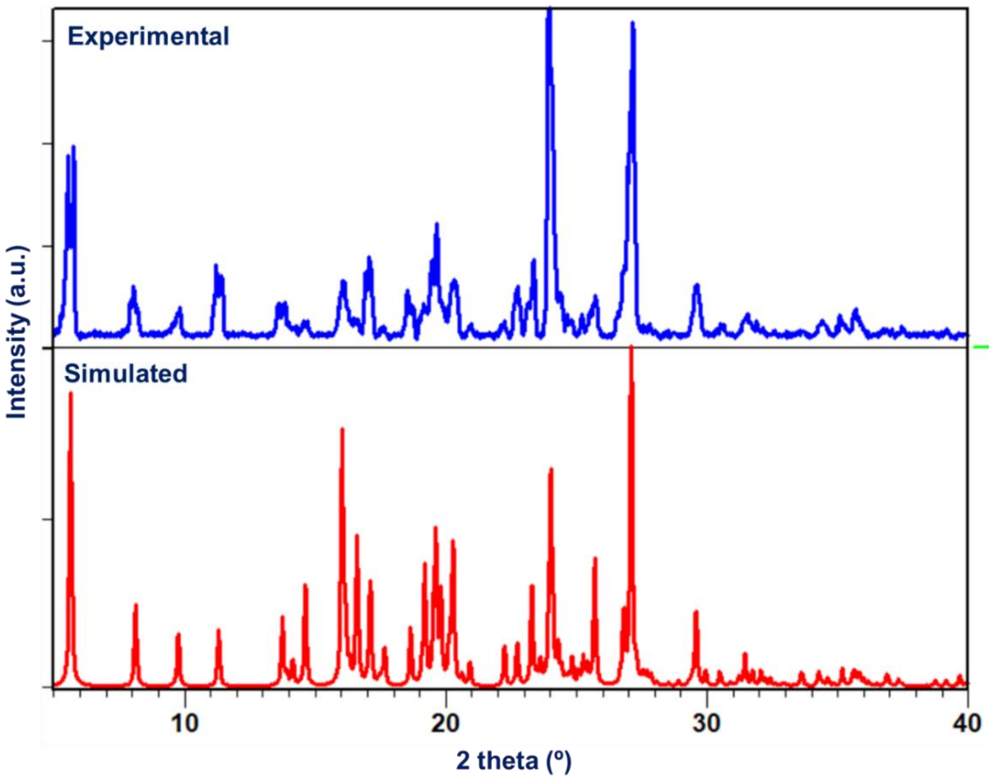

Figure S10: PXRD data of **Bza-1-Form I** obtained through solution crystallization from nitromethane.

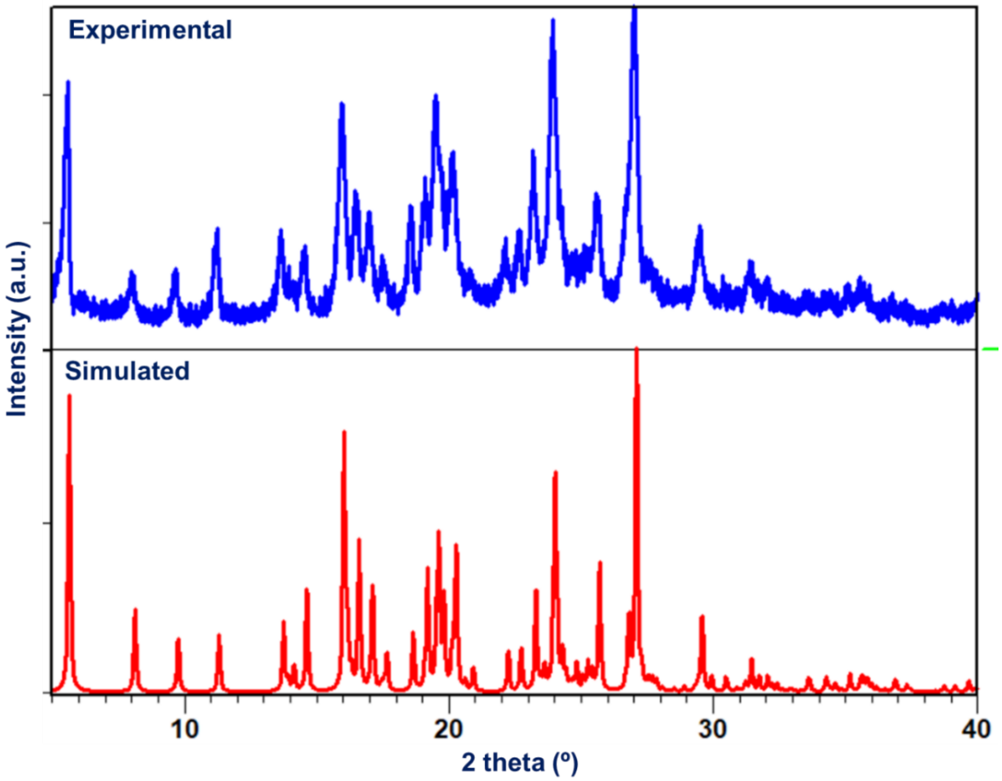

Figure S11: PXRD data of **Bza-1-Form I** obtained through solution crystallization from ACN.

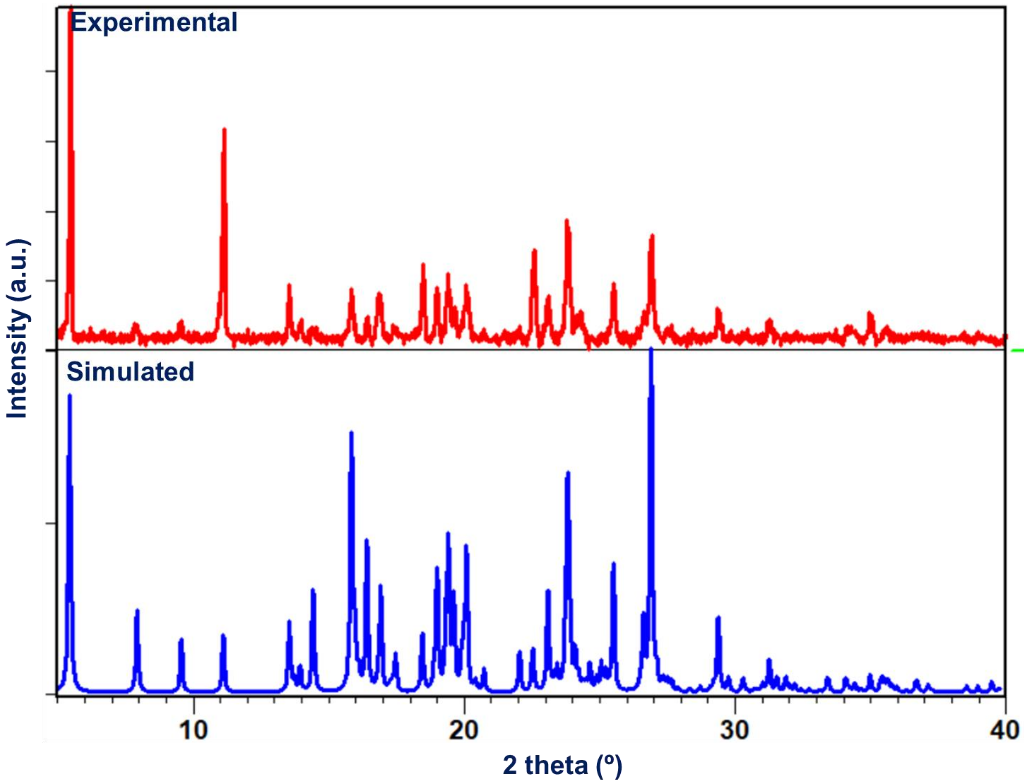

Figure S12: PXRD data of **Bza-1-Form I** obtained through solution crystallization from methanol.

Investigation of competitive equilibrium: Solution crystallization with varying percentages of benzylamine in methanol:

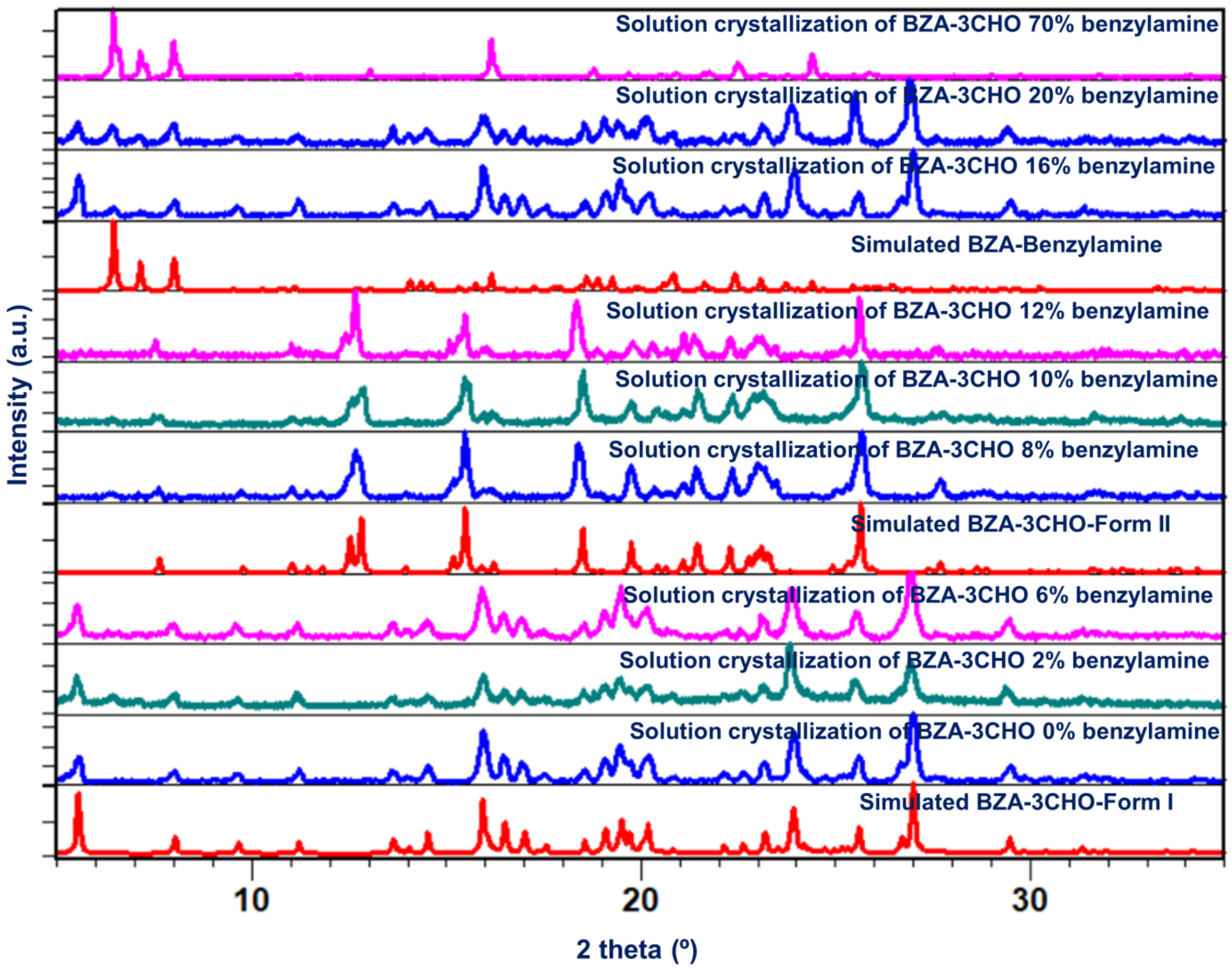

Figure S13: PXRD pattern comparison of solution crystallization with varying percentages of benzylamine in methanol.

Solution crystallization using 10% benzylamine solution of different solvents: 10 µL (0.1 mmol) of benzylamine was dissolved in 2000 µL of different solvents, i.e., DCM, toluene, dioxane, ACN, ethyl acetate. 0.1 mmol of benzilic acid (22.3 mg) and 0.1 mmol of (E)-3-(2-(pyridine-4-yl) vinyl) benzaldehyde (20.9 mg) were weighed in five different glass vials and dissolved in 200 µL of the prepared solvents. The solution was kept for slow evaporation, and the obtained crystals were ground and used for Powder X-ray diffraction.

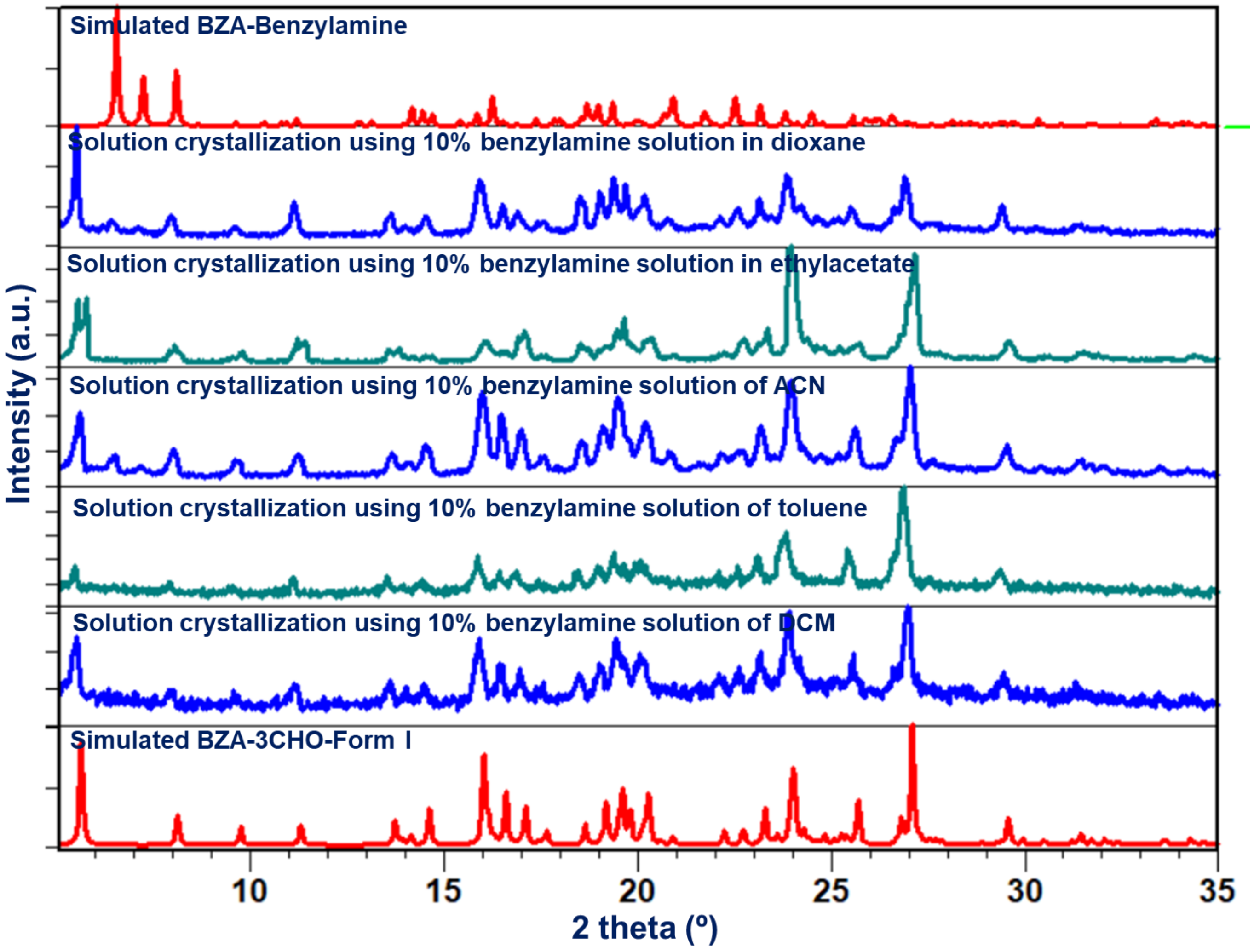

Figure S14: PXRD data of solution crystallization with 10 percentages of benzylamine in different solutions.

Bza-1-Form II:

Obtained through solution crystallization from 8% benzylamine methanol solution.

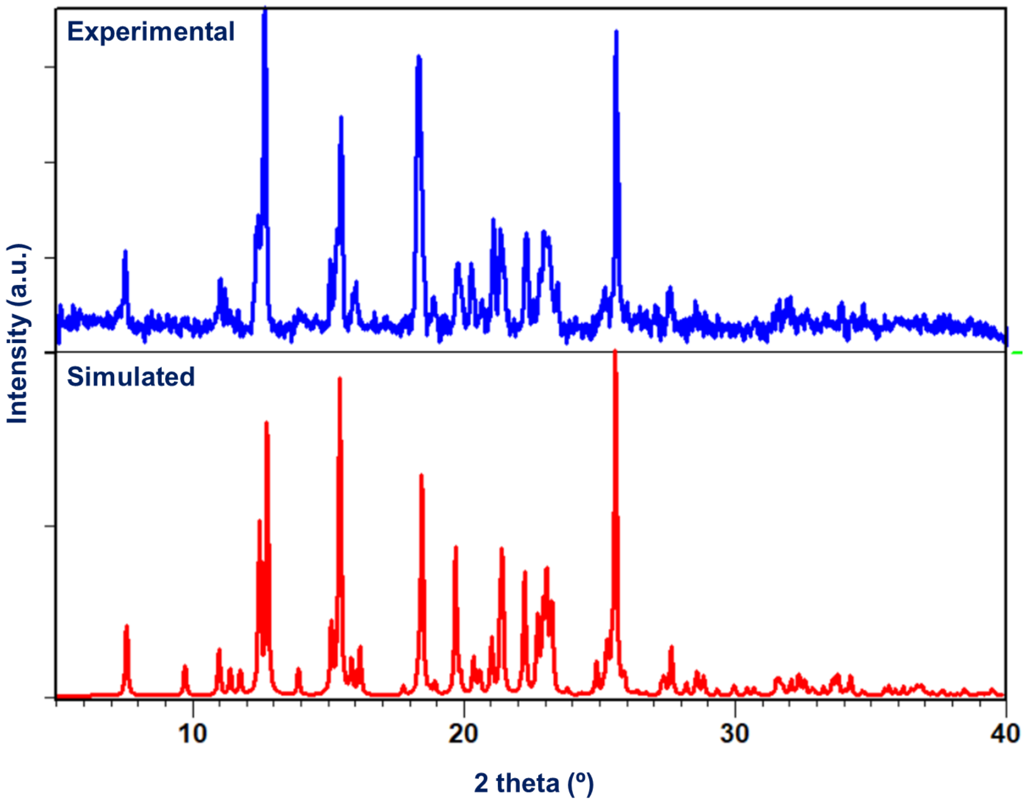

Figure S15: PXRD data of **Bza-1-Form II** obtained from 8% benzylamine in methanol.

Obtained through solution crystallization from 10% benzylamine methanol solution.

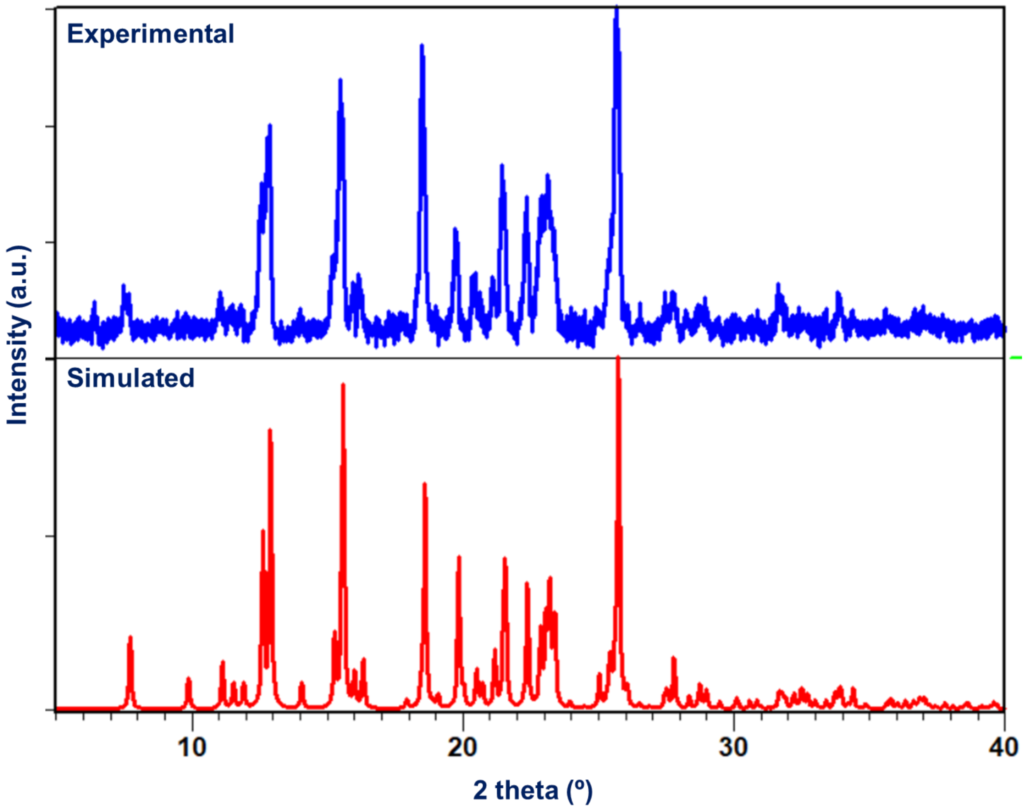

Figure S16: PXRD data of **Bza-1-Form II** obtained from 10% benzylamine in methanol.

S4. Additional DSC analysis:

Supplementary Note 3

(a) Bza-1-Form I: The PXRD-matched sample of Form I was used for the DSC analysis.

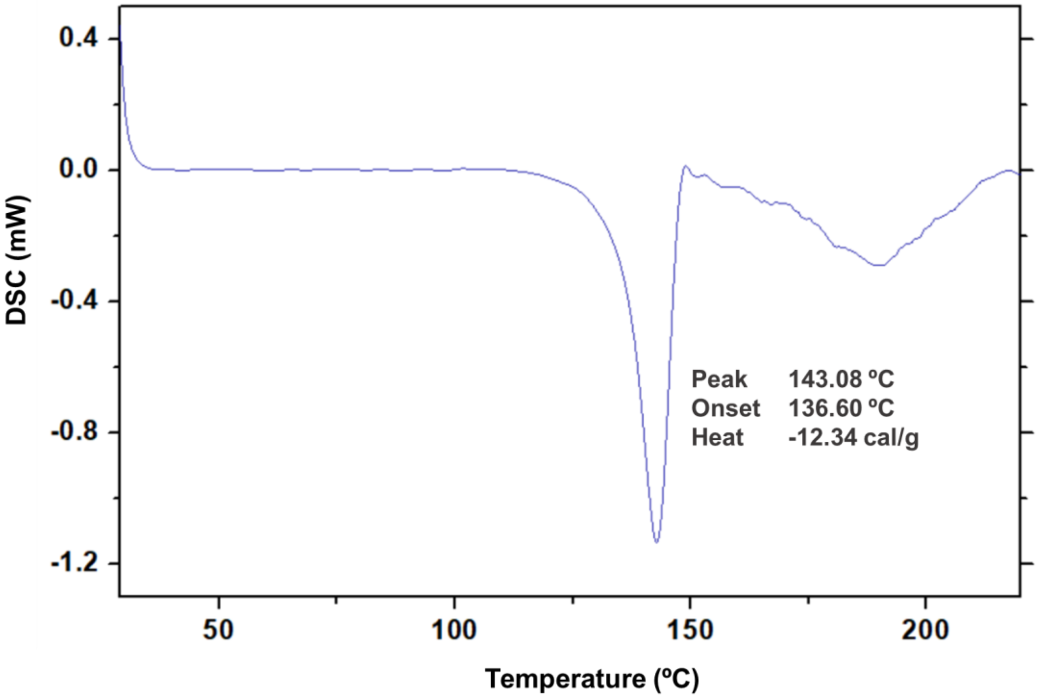

Figure S17: DSC data of **Bza-1-Form I.**

Bza-1-Form II: The PXRD-matched sample of Form II was used for the DSC analysis.

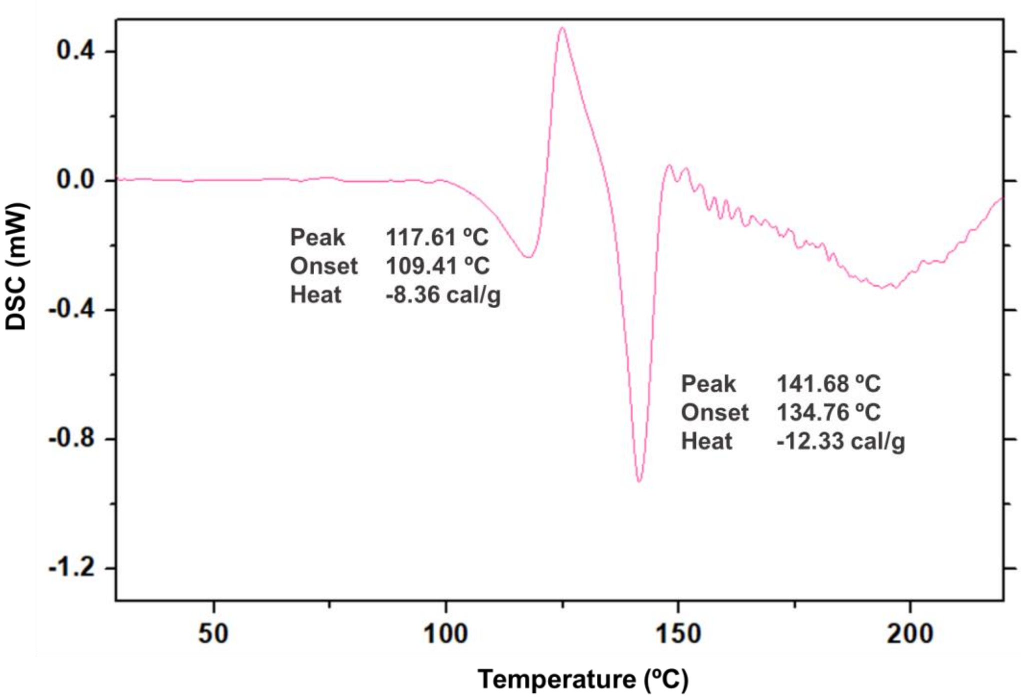

Figure S18: DSC data of **Bza-1-Form II.**

S5. Additional NMR analysis:

Supplementary Note 4

Native coformer (1) Before and after irradiation:

The purified powder sample, obtained through column chromatography and subsequent solvent evaporation, was irradiated under broadband UV radiation for 12 hours. The irradiated sample was dissolved in $CDCl_3$ for 1H NMR, and the same sample was used for ^{13}C NMR data collection. While the irradiated sample was compared with the native sample through NMR, no new peaks were observed in the irradiated sample, suggesting the photo inactivity of the native compound.

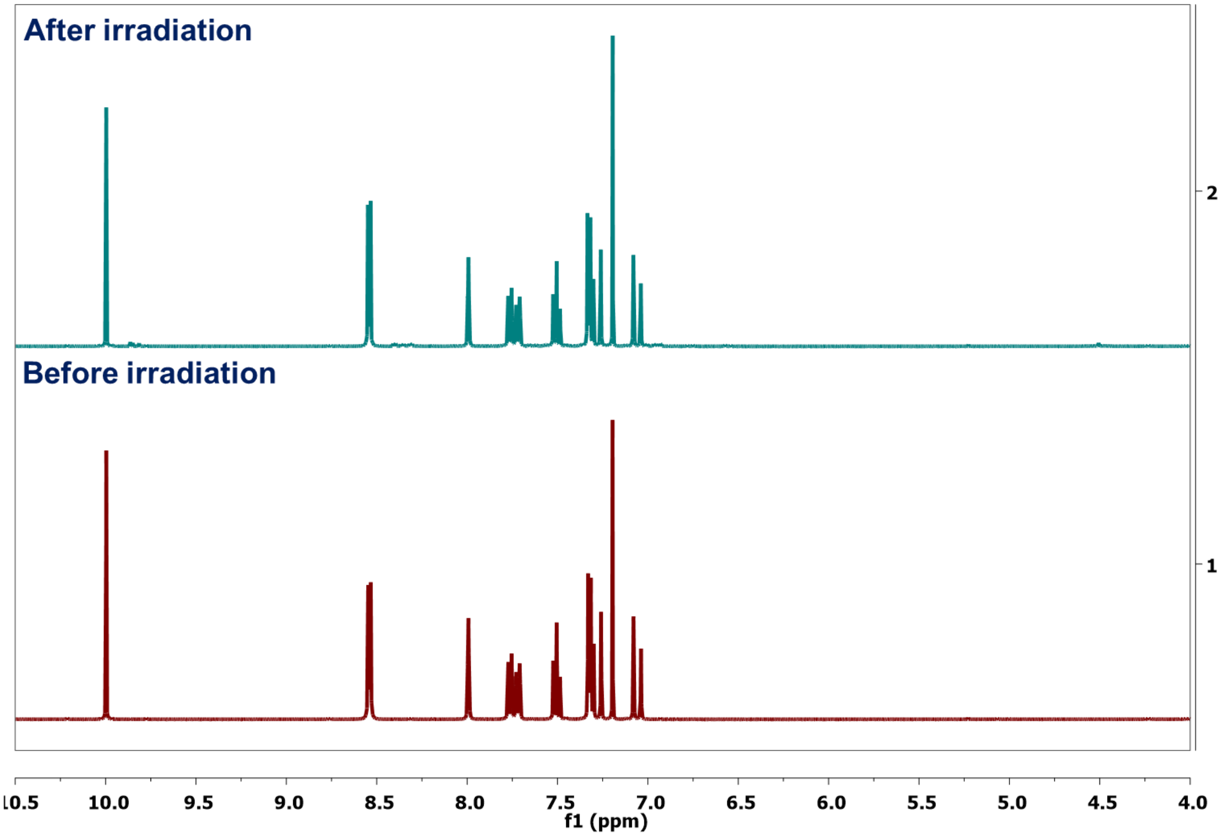

Figure S19: Stacked ^{1}H NMR spectra of **1** before and after irradiation.

^{13}C NMR spectra of before and after irradiated native compound (1):

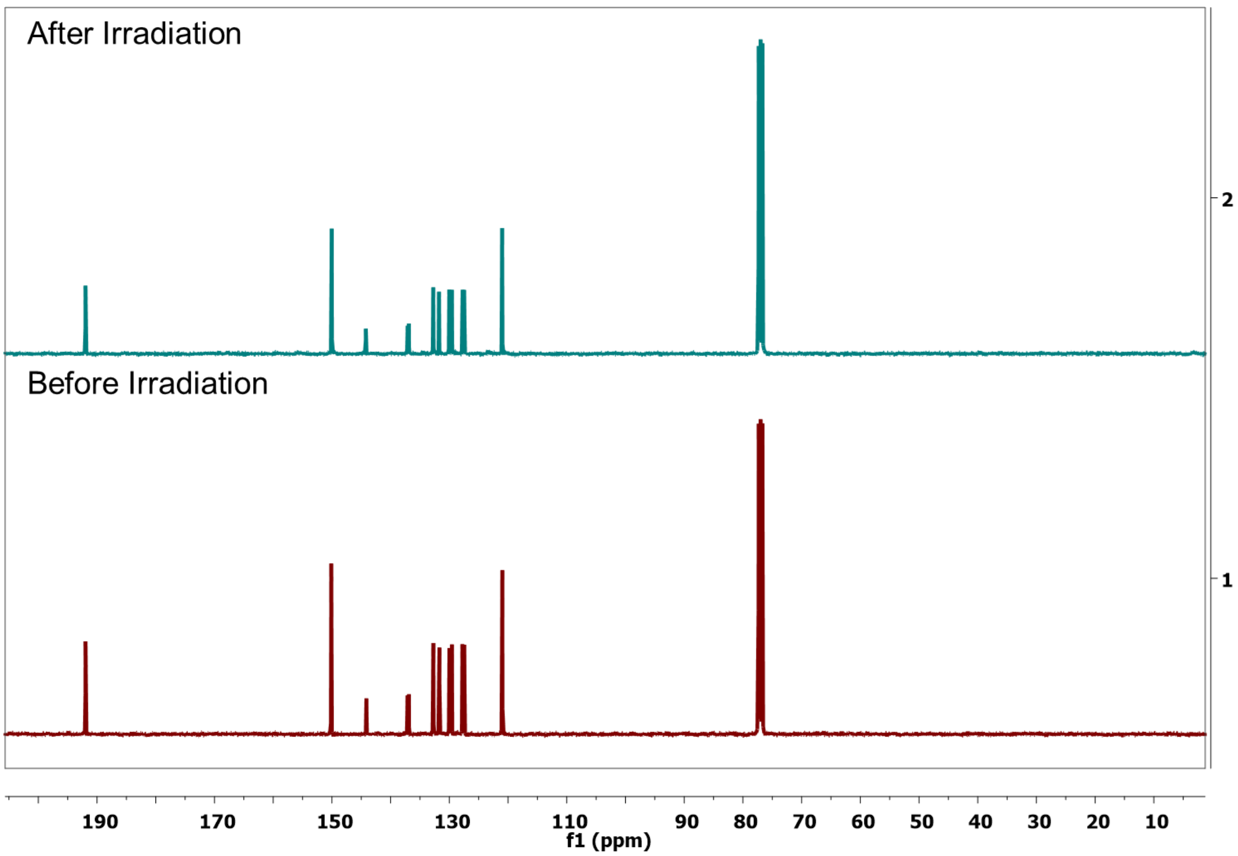

Figure S20: Stacked ^{13}C NMR spectra of **1** before and after irradiation.

Bza-1-Form I: Before and after irradiation:

After 4 hours and 12 hours: The PXRD-matched powder sample of Form I was irradiated for 4 hours and dissolved in $CDCl_3$ to collect the NMR.

(i) The appearance of new peaks at 8.47-8.59 ppm suggested the photoreactive nature of the sample.

(ii) Along with that, new peaks at 6.69-6.72 ppm (and 6.91-6.94 with coupling constants of 12.56 and 12.26 Hz, suggest the formation of a cis isomer.

(iv) Integration of reacted and non-reacted protons and comparison of the same suggest an ~18% cis isomer formation upon broadband UV radiation.

(v) Similarly, when the reactant and product proton peak were integrated for 12 hours of irradiated samples, comparing with unreacted sample peaks suggest ~42% conversion to the cis product.

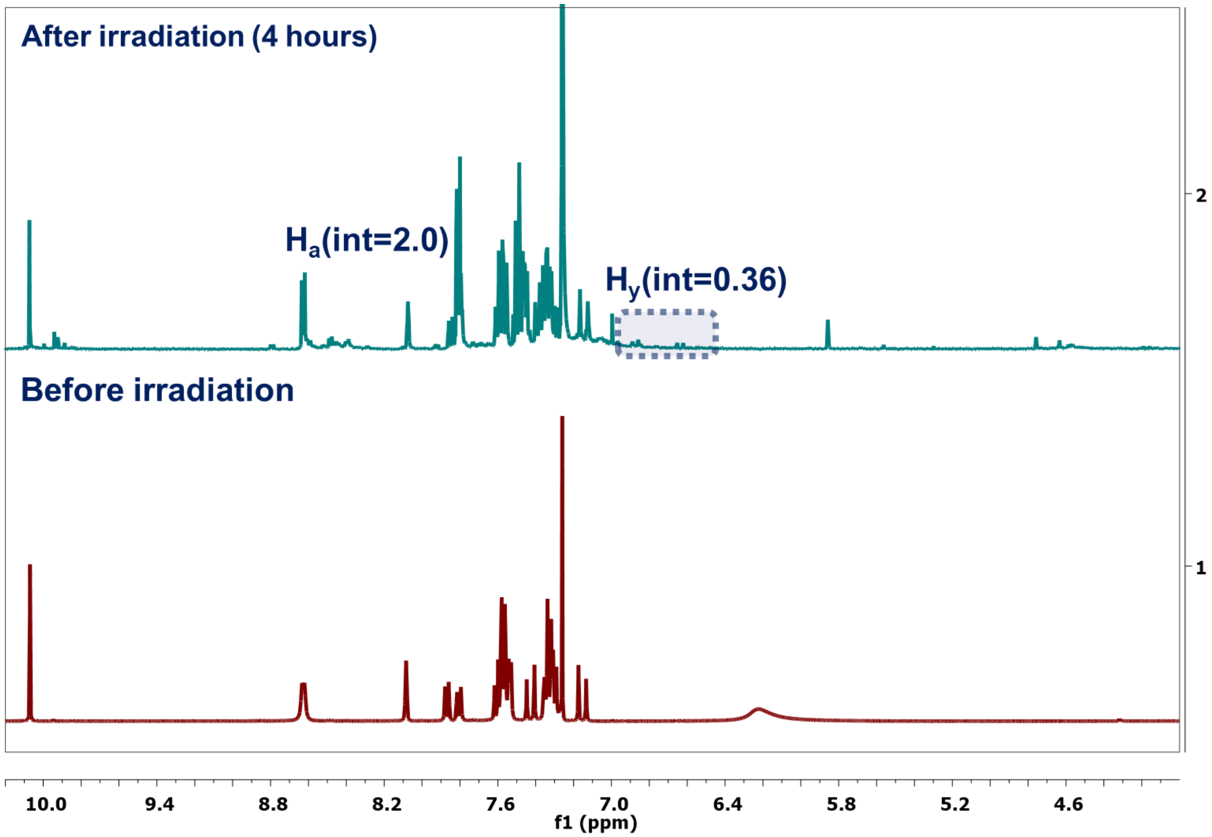

Figure S21: Stacked ^{1}H NMR spectra of **Bza-1-Form I** before and after irradiation (4 hours).

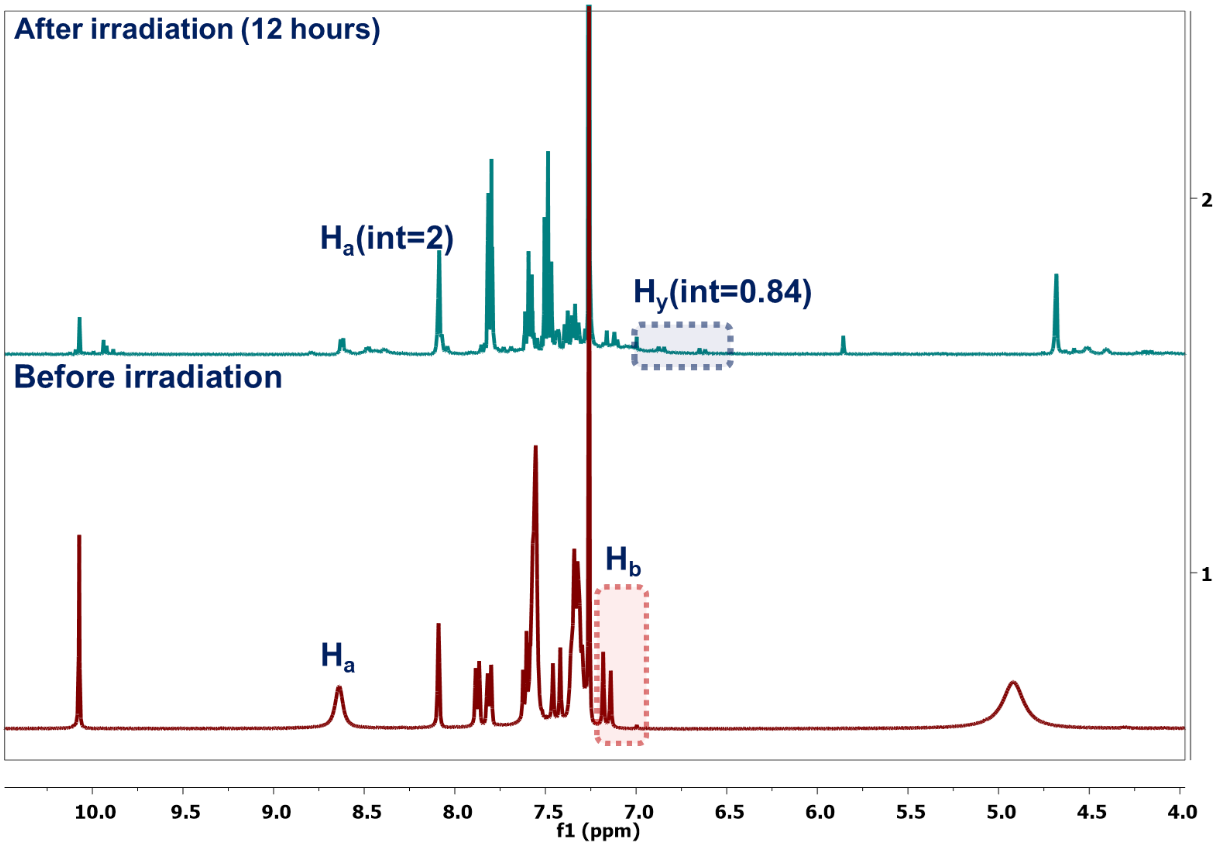

Figure S22: Stacked ^{1}H NMR spectra of **Bza-1-Form I** before and after irradiation (12 hours).

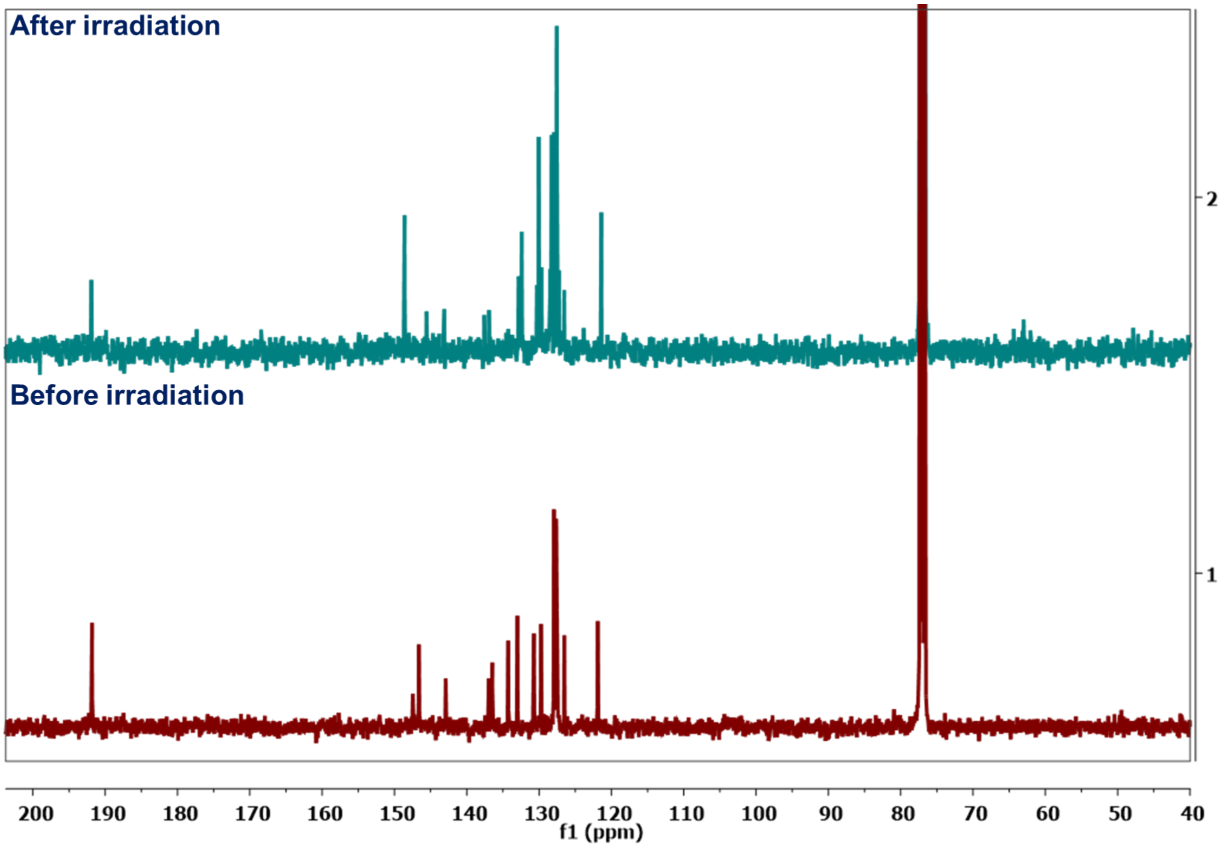

Figure S23: Stacked ^{13}C NMR spectra of **Bza-1-Form I** before and after irradiation (12 hours).

Bza-1-Form II: Before and after irradiation: The PXRD-matched Form I samples were irradiated for 10 and 12 hours, and the irradiated samples were dissolved in $CDCl_3$ and used for NMR analysis.

(i) A new peak appeared at 8.31-8.33 ppm, suggesting the photo-reactive nature of the sample.

(ii) Another new peak appeared in the aliphatic region around 4.50-4.55 ppm, implying the formation of photodimer.

(iii) The integration of 4.50-4.55 ppm and 8.31-8.33 was the same, suggesting this peak corresponds to the ortho pyridine proton of the photo-dimer.

(iv) The integration of reactant and product proton peak suggested a formation of 93% cyclobutane in the 10-hour irradiated sample.

(v) In the case of a 12-hour irradiated sample, the reactant proton peak completely disappeared, suggesting a complete conversion to the product.

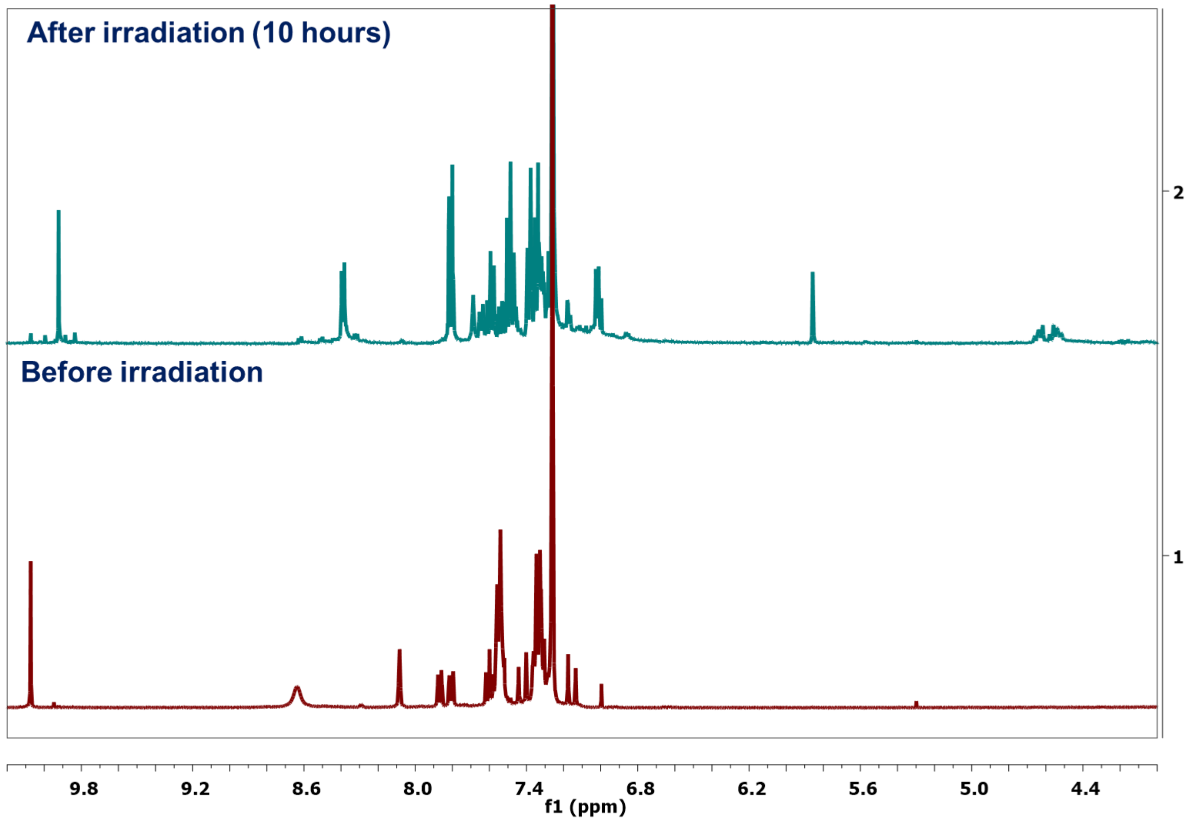

Figure S24: Stacked ^{1}H NMR spectra of **Bza-1-Form II** before and after irradiation (10 hours).

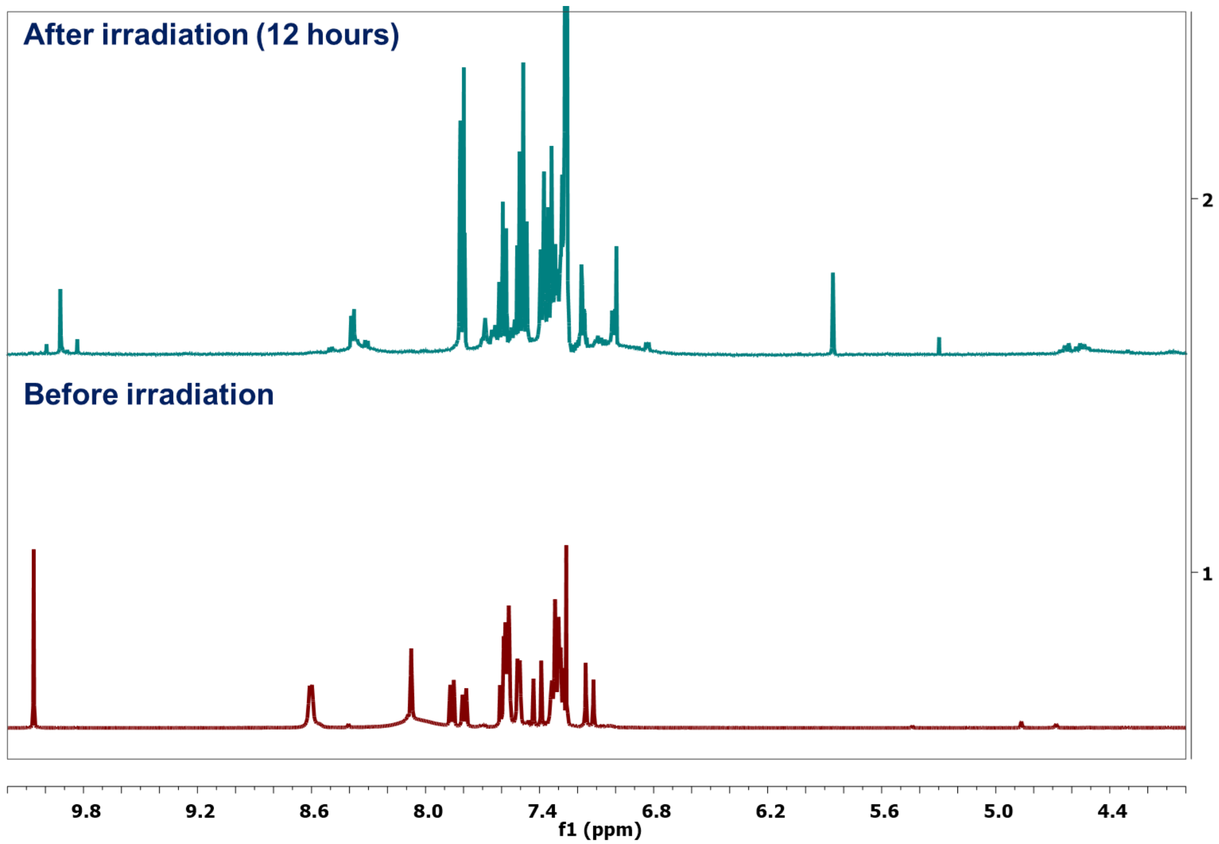

Figure S25: Stacked ^{1}H NMR spectra of **Bza-1-Form II** before and after irradiation (12 hours).

^{13}C NMR spectra of before and after irradiated Bza-1-Form II: The appearance of a new peak around 45 ppm in ^{13}C NMR also confirmed the formation of the cyclobutane photoproduct.

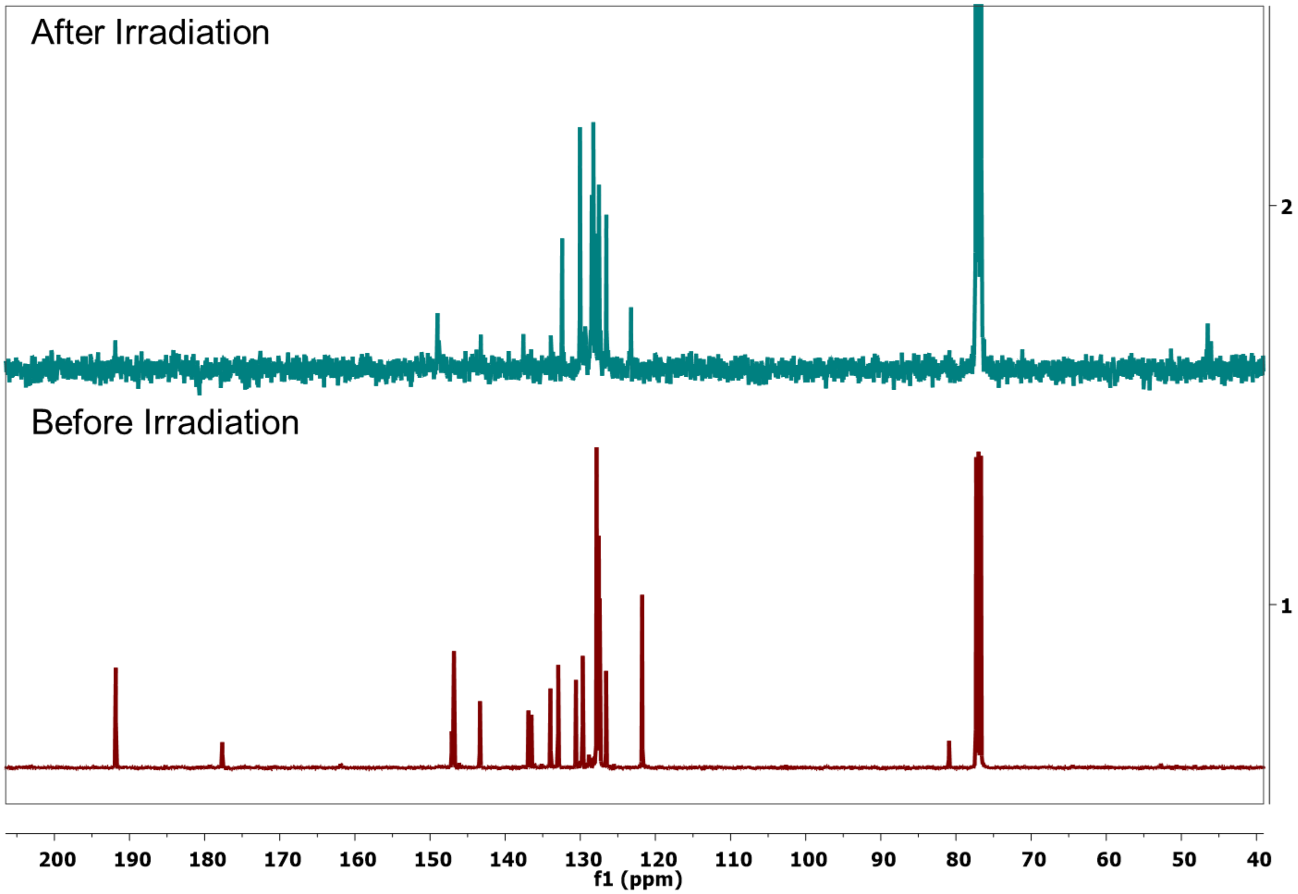

Figure S26: Stacked ^{13}C NMR spectra of **Bza-1-Form II** before and after irradiation (12 hours).

Comparative ^{1}H NMR analysis: To get more insights into this, ^{1}H NMR spectra of Bza-1-Form I (10 mg) was collected in three different experimental conditions (i) before irradiation (ii) after 8 hours of irradiation, (iii) the sample was dissolved in $CDCl_3$ and the solution was subsequently irradiated for 8 hours. In the case of (i), no changes were observed. In (ii), 28% cis product formation could be identified from the 1H NMR spectrum with respect to unreacted samples. In the case of (iii), the complexity of the ^{1}H NMR spectrum indicates multiple product formation, which was difficult to decipher.

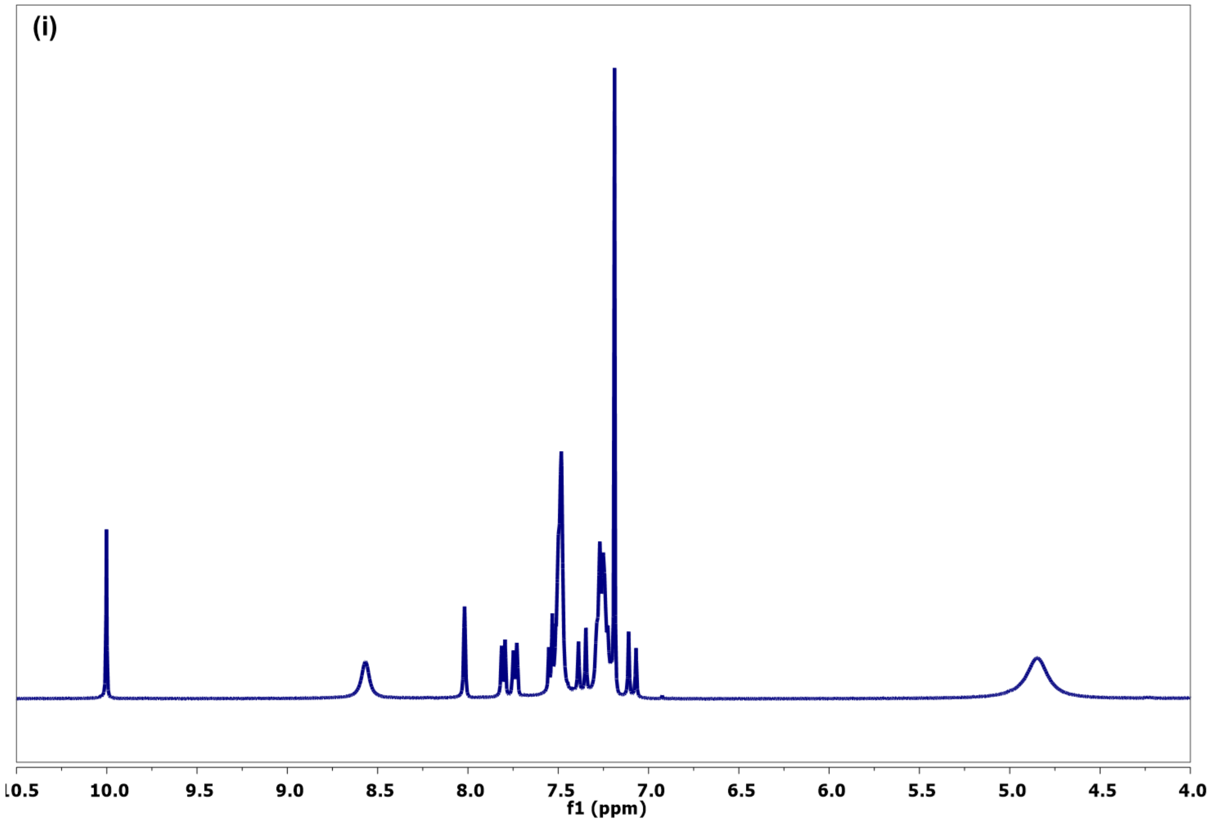

Figure S27: ^{1}H NMR spectra of **Bza-1-Form I** before irradiation.

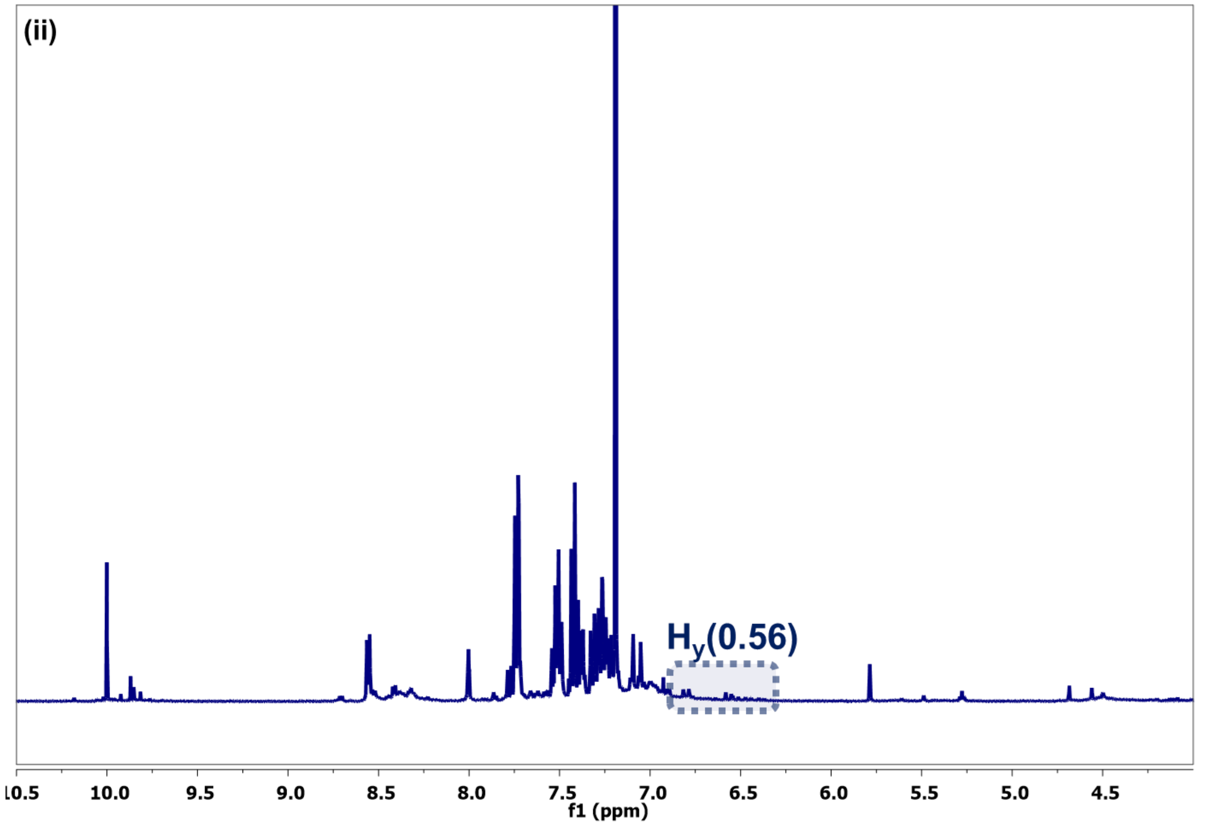

Figure S28: ^{1}H NMR spectra of **Bza-1-Form I** after irradiation (8 hours).

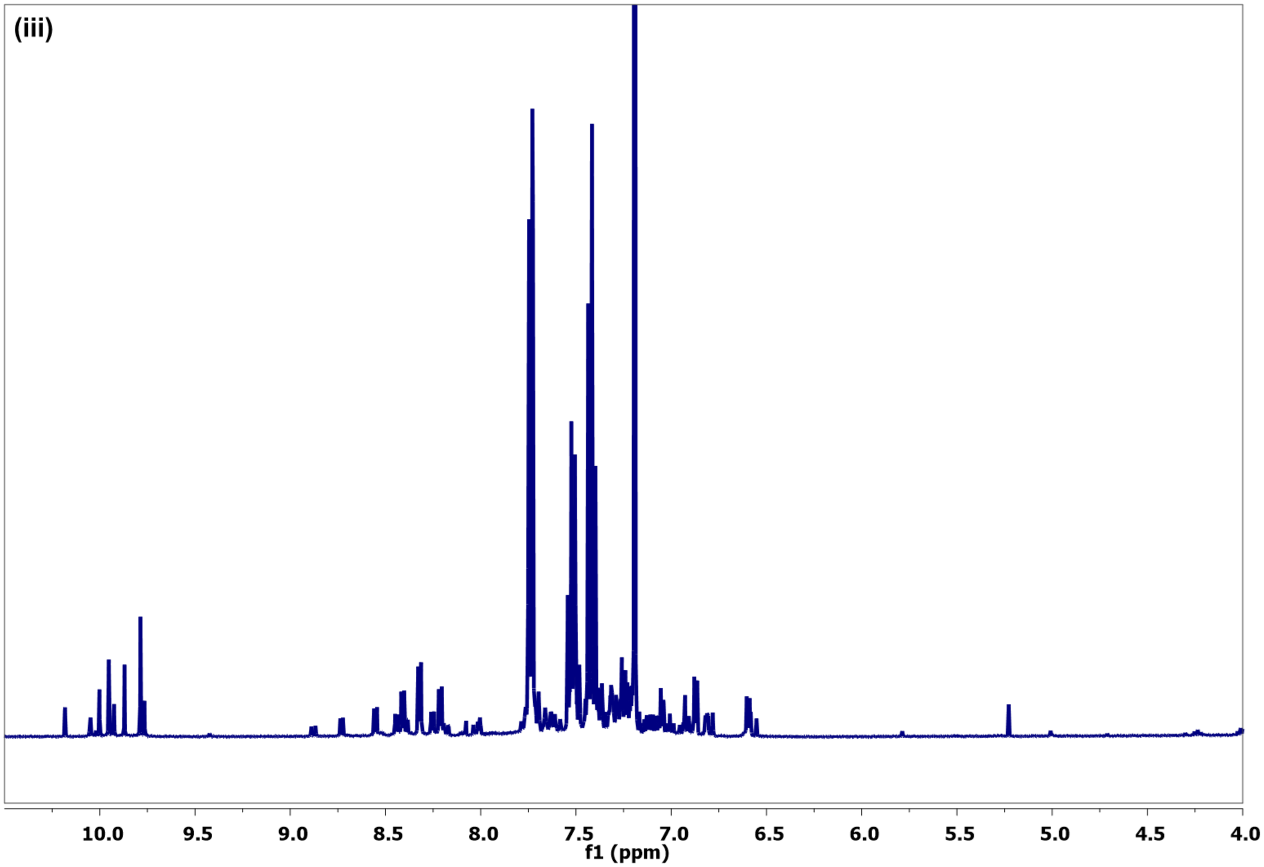

Figure S29: ^{1}H NMR spectra of **Bza-1-Form I** irradiated by dissolving in $CDCl_3$ (8 hours).

^{1}H NMR analysis of a representative imine exchange reaction:

(i) 10.7 mg benzylamine was dissolved in 2.5 ml of $CDCl_3$. 22.5 mg of **1** was dissolved 500 µl of the prepared solution and used for ^{1}H NMR analysis.

The appearance of a new peak (H3) at 8.35 ppm (1H, singlet) suggested a possible imine formation and another new peak appeared (H_1) at 8.49-8.50 ppm (2H, doublet, J=6hz) also corroborates well with the imine formation. The disappearance of benzylamine amine peak (H_p) suggested a complete consumption of benzylamine. Apparently, a new peak appeared downfielded compared to the benzylamine CH2 (Hp) proton peak at 4.77 ppm (H_4, 2H Singlet). The relative integration of H4 with H1, and H_3 (2:2:1) confirmed the complete conversion of benzylamine to the respective imine product.

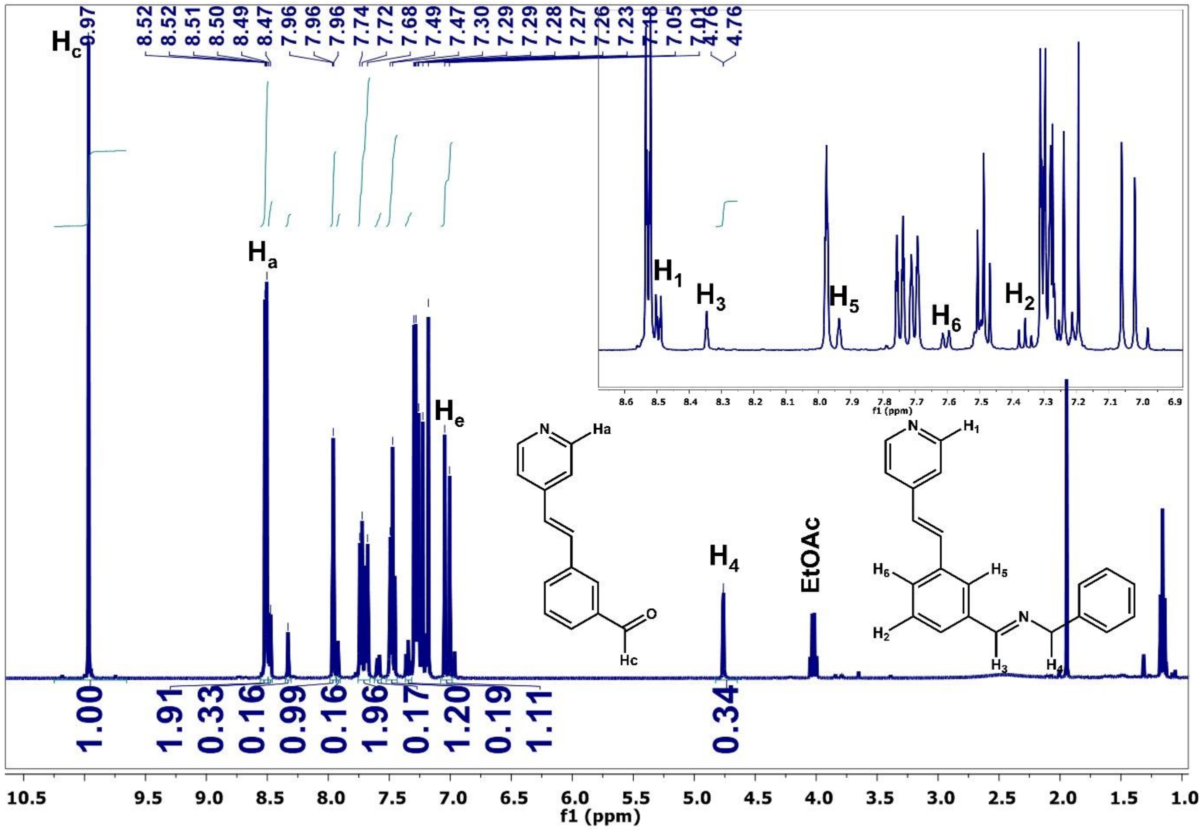

Figure S30: ^{1}H NMR spectra **of 1+benzylamine.** Formation of the respective imine is shown in the figure.

(ii) In another experiment, 10.7 mg benzylamine was dissolved in 2.5 ml of $CDCl_3$. In 500 µl of the prepared solution 22.3 mg Bza and 21.0 mg **1** was added together and the resultant mixture was used for NMR analysis.

The appearance of H_3 and H_4 Peaks confirmed the formation of the respective imine. However, another peak was observed at 4.58 ppm, which is downfielded compared to benzyl amine CH_2 proton peak H_p (3.78 ppm) and upfielded compared to H_4 proton peak. This peak is possibly corresponding to the benzylamine salt, formed in presence of Bza. This NMR data suggested a possible equilibrium exists between benzylamine salt formation vs imine formation in presence of Bza, at the given concentration and solvent conditions. A stacked NMR of **1** in benzylamine solution, Bza-1 in benzyl amine solution and benzylamine is given below.

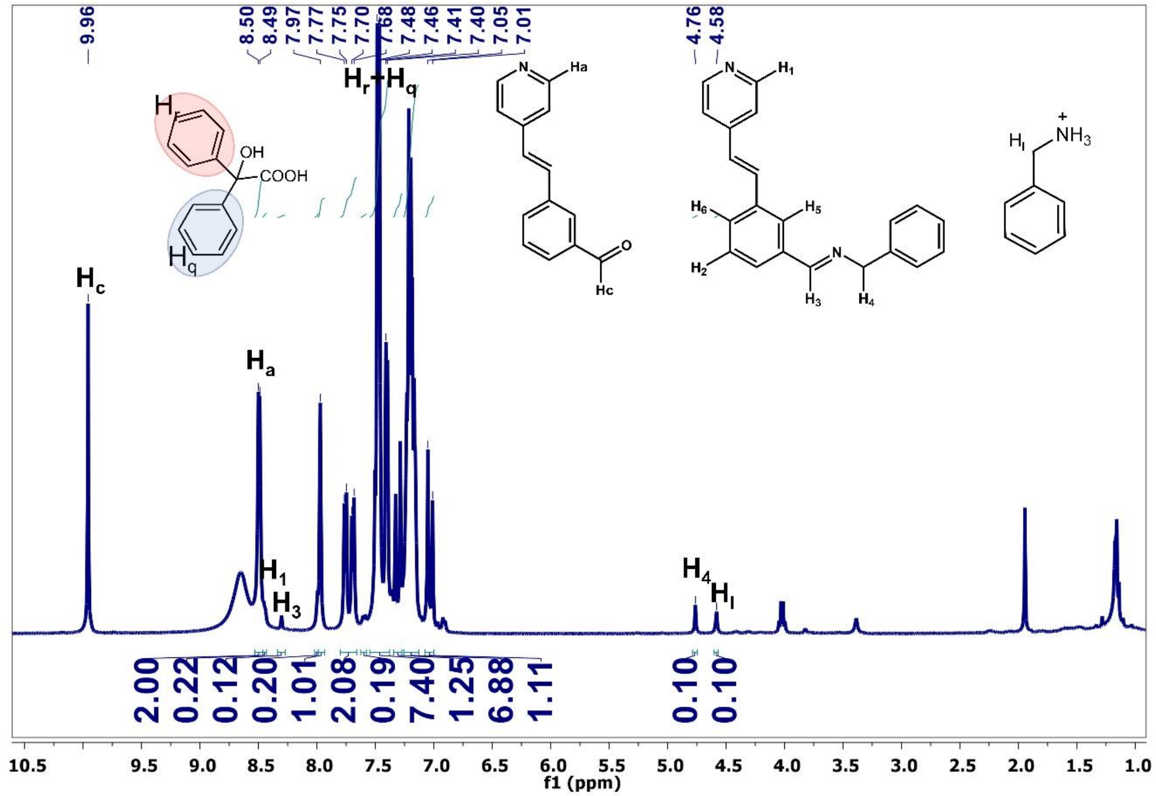

Figure S31: ¹H NMR spectra of **Bza+1+benzylamine.** Formation of the respective imine as well as the Bza-benzylamine salt is shown in the figure.

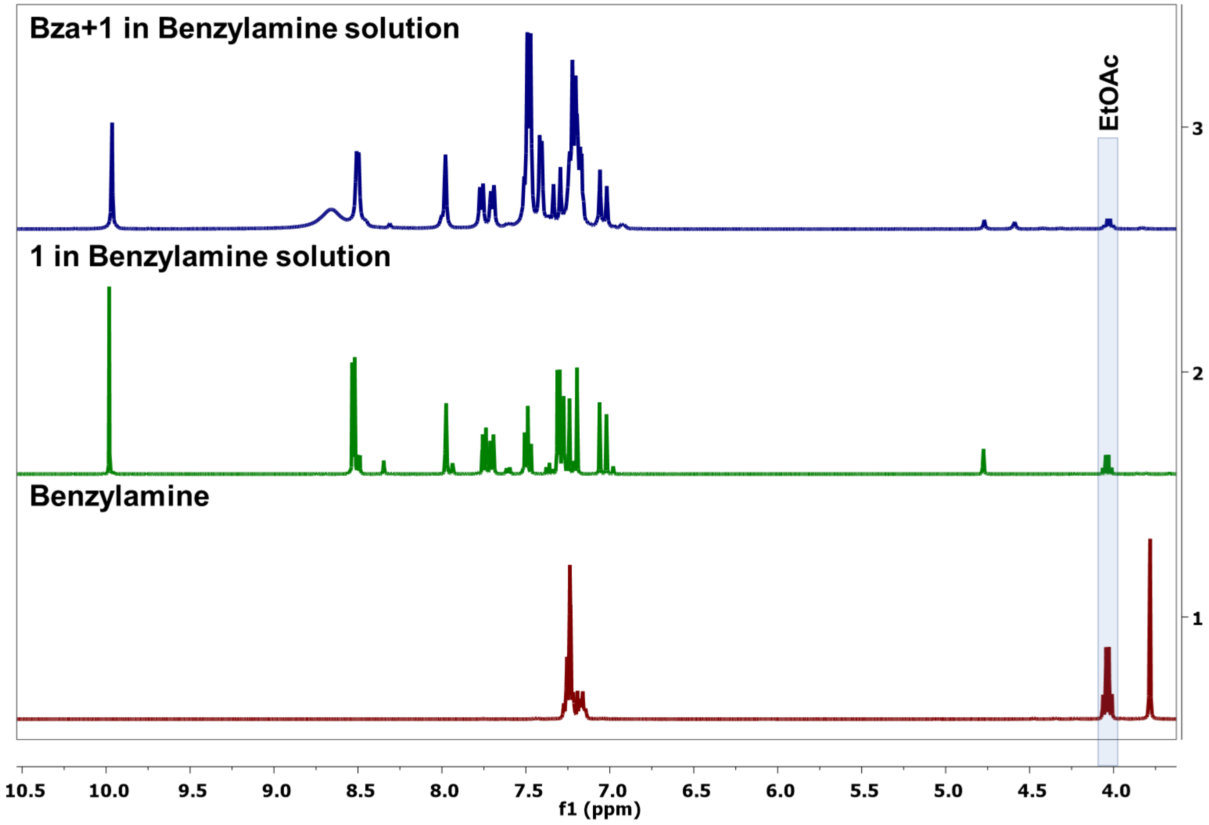

Figure S32: Stacked ¹H NMR spectra of **benzylamine, 1+benzylamine and Bza+1+benzylamine.**

S6. Additional Mass Spectra (LC-MS):

Supplementary Note 5

Form I: Single crystals of Bza-1-Form I crystals were irradiated, and single crystals of before and after irradiated samples were dissolved in methanol and used for LC-MS data collection. The m/z=210 corresponds to the native **1**. No traces of dimer peaks were observed for the irradiated sample, which suggested no photochemical conversion to dimer.

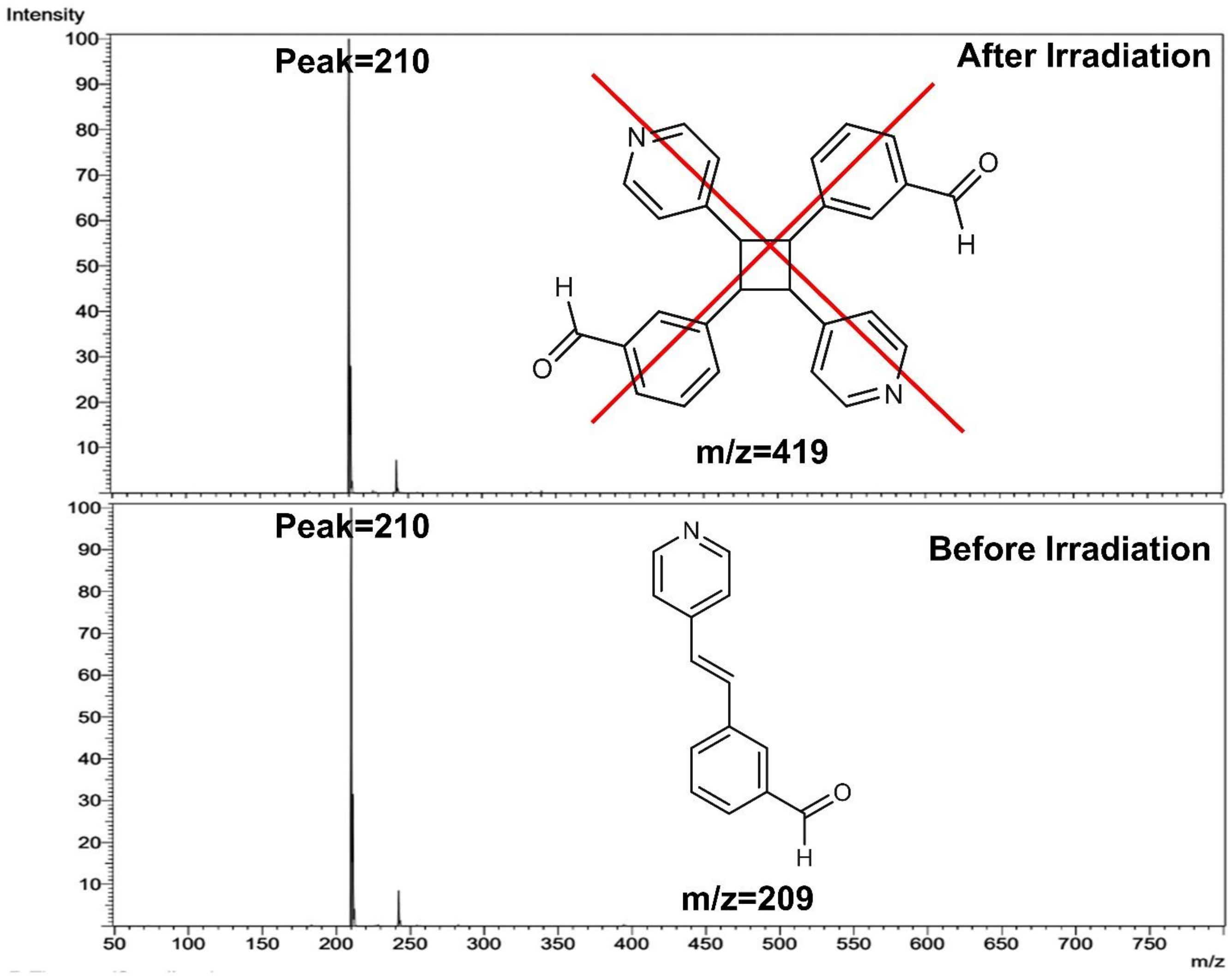

Figure S33: Stacked LCMS spectra of **Bza-1-Form I** before and after irradiation.

Form II: Similarly, the single crystals Bza-1-Form II were irradiated under broadband UV radiation, and the irradiated sample was dissolved in methanol and used for LC-MS. The m/z=210 peak had appeared for the native compound, and with that, a new peak at m/z=419 had appeared due to the formation of the photodimer.

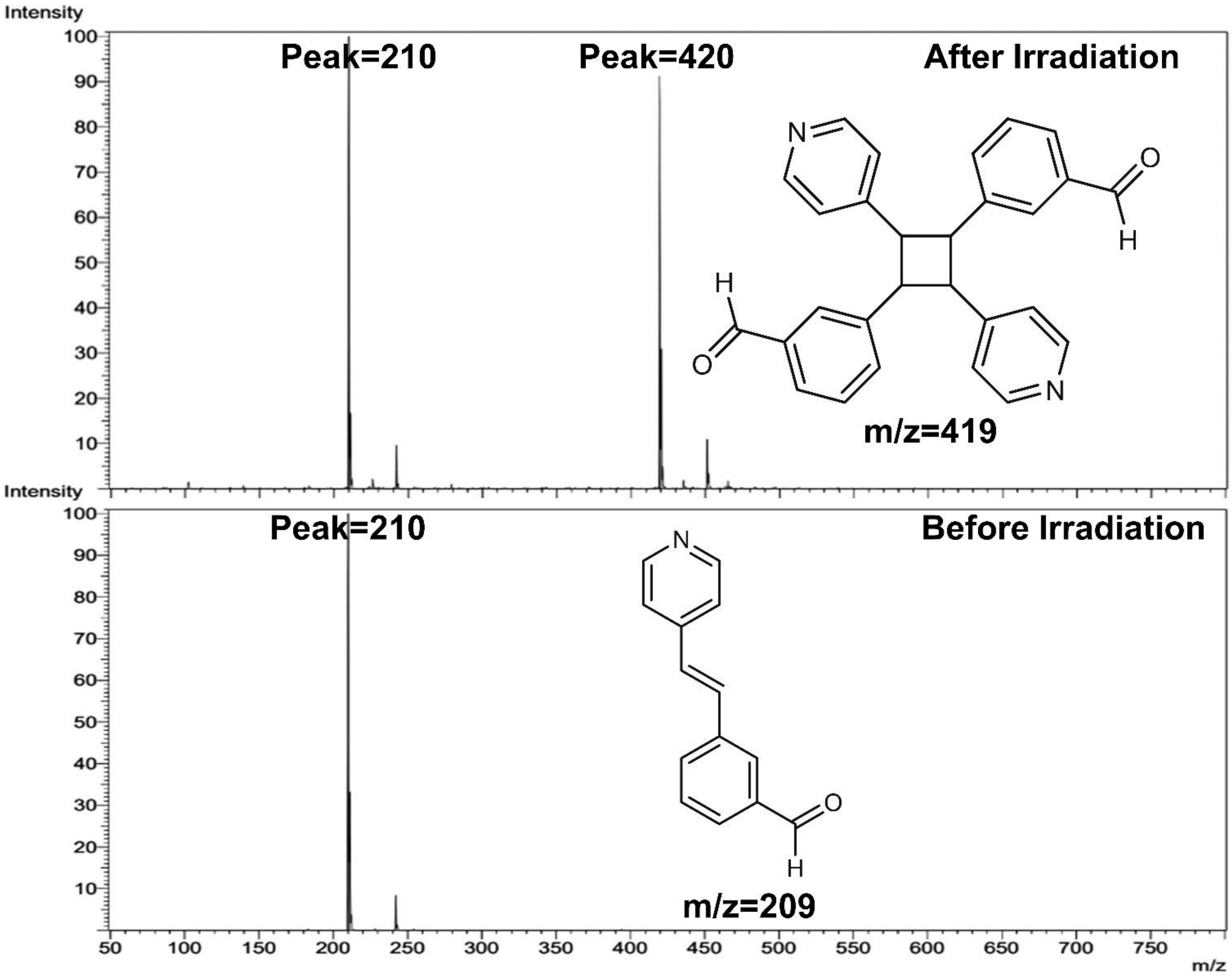

Figure S34: Stacked LCMS spectra of **Bza-1-Form II** before and after irradiation.

S7. Computational Calculations:

Supplementary Note 6

Electrostatic Surface Potential (ESP):

ESP charge for the molecule was calculated using Orca,[1] Multiwfn,[2] and VMD[3] software. The molecule was visualized using Avogadro software, and the geometry of the molecule was optimized using Orca with the keyword ALLPOP KEEPDENS to generate the surface charges. The molecule's surface was generated using Multiwfn[2] software and visualized through VMD[3] software.

Maxima: 29.92 kcal/mol

Minima: -38.37 kcal/mol

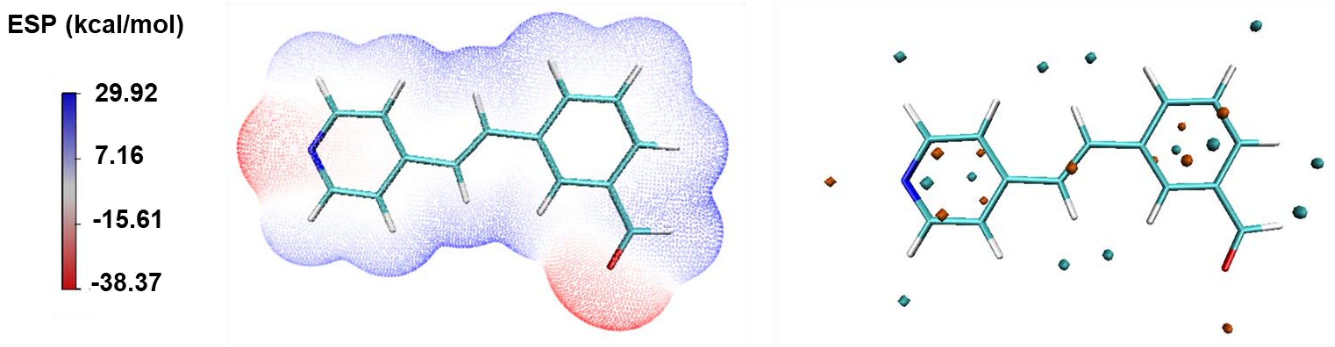

Figure S35: Electrostatic Surface Potential (ESP) of **1.**

HOMO-LUMO calculation:

HOMO-LUMO of the cocrystal system was computed employing the crystallographic geometry. The single-point energy of the resultant crystals was determined using the Gaussian 09[4] software. The single point energy calculation was done using the Hybrid DFT-based approach B3LYP/6311(d, p) G basis set; no additional optimization was done. The structure and the HOMO and LUMO were visualized using Gauss View. In Both calculations, the HOMO was formed on the Bza molecule, and the LUMO was comprised of **1.**

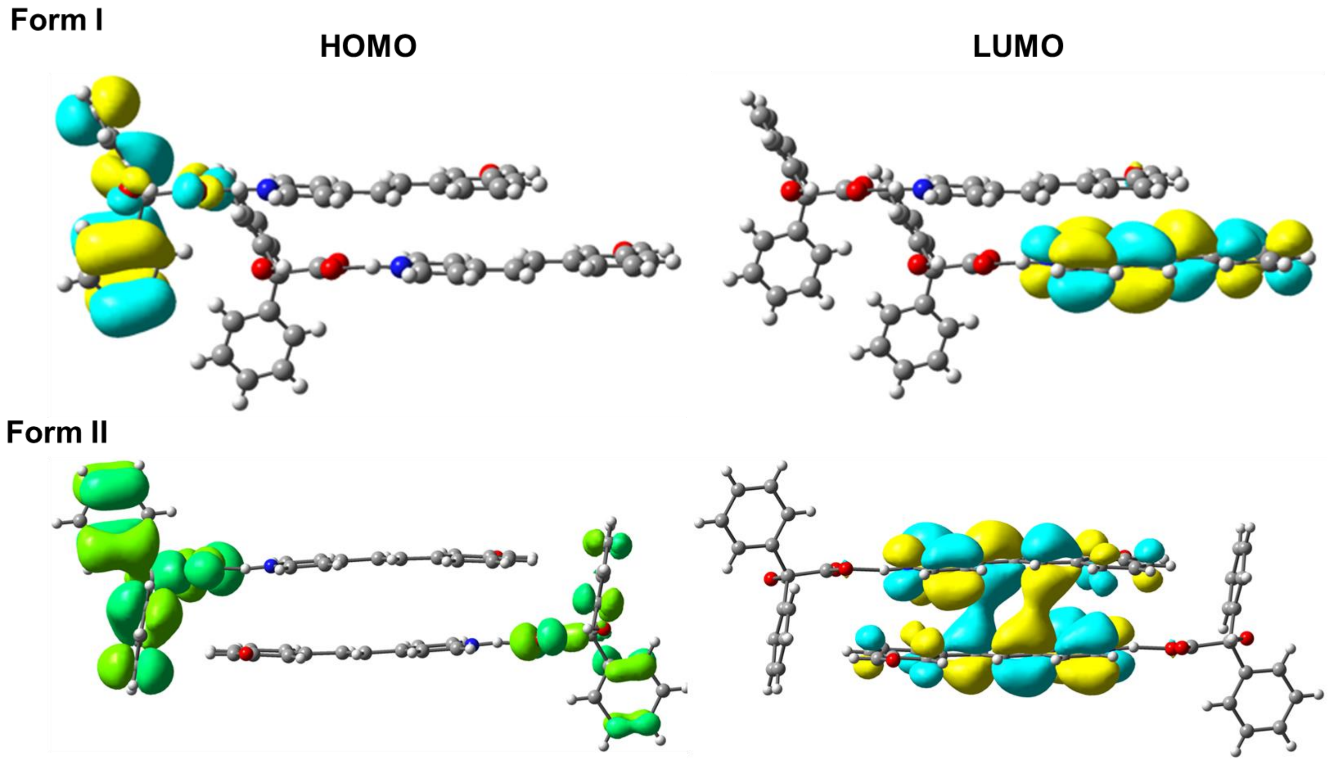

Figure S36: Calculated HOMO and LUMO of **Bza-1 Form I and Bza-1 Form II.**

S8. Photophysical properties:

Supplementary Note 7

(a) Absorption Spectra:

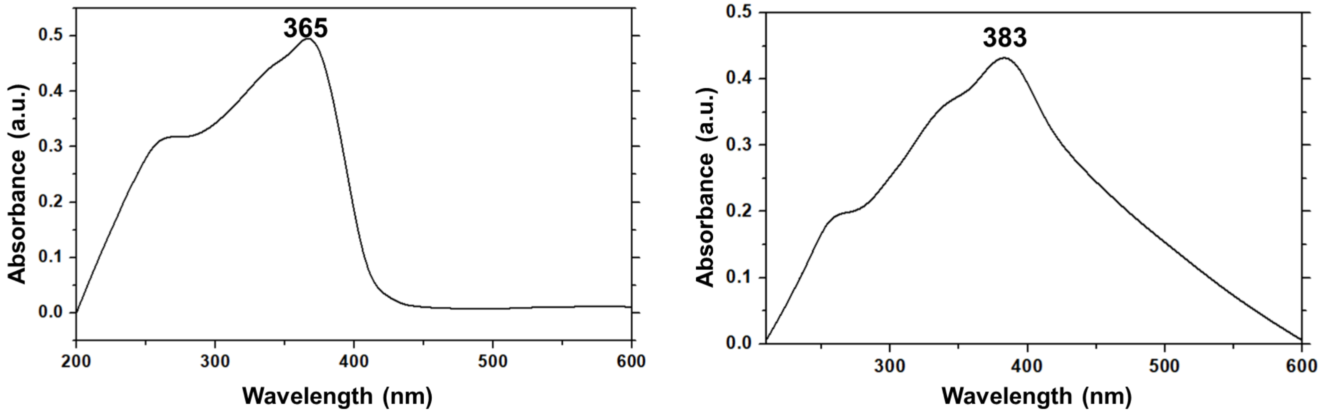

Figure S37: Absorption spectra of **Bza-1 Form I and Bza-1 Form II.**

(b) Emission Spectra:

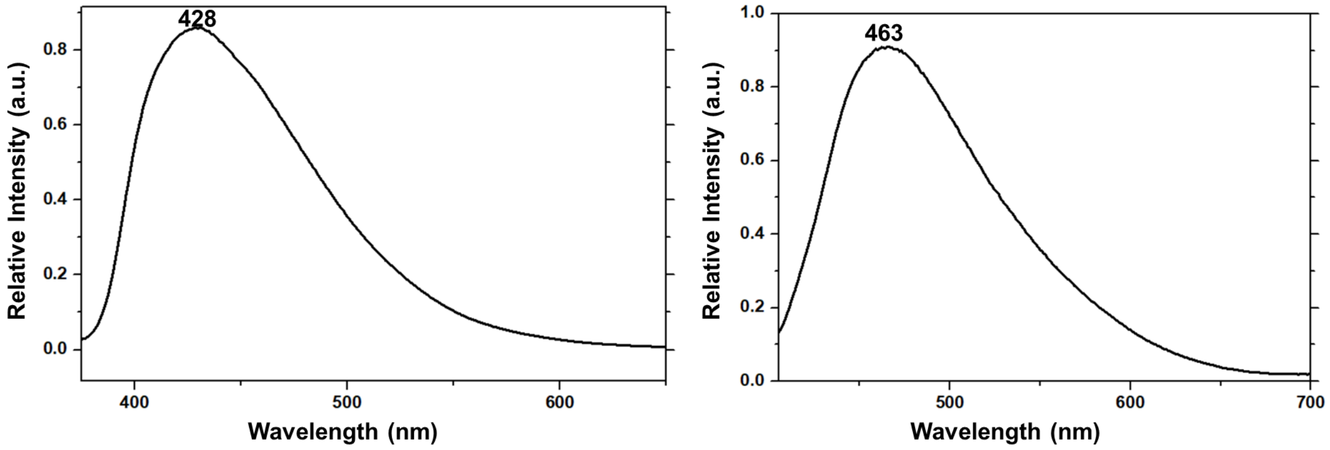

Figure S38: Bza-1 Form I and Bza-1 Form II.

Table 2: Photophysical properties of Bza-1-Form I and Bza-1-Form II.

System	Excitation Maxima (nm)	Emission maxima (nm)	Lifetime (ns)
**Bza-1-Form I**	365	428	0.0713712
**Bza-1-Form II**	383	463	0.172787

S9. Supplementary References:

1. Neese, F. The ORCA program system. *WIREs Comput Mol Sci* **2**, 73–78 (2012).

2. Lu, T. & Chen, F. Multiwfn: A multifunctional wavefunction analyzer. *J Comput Chem* **33**, 580–592 (2012).

3. Humphrey, W., Dalke, A. & Schulten, K. VMD: Visual molecular dynamics. *Journal of Molecular Graphics* **14**, 33–38 (1996).

4. M. J. Frisch, *et al.*, Gaussian 09 (Gaussian, Inc., Wallingford CT, 2009).
