# Supplementary material for: Regulating photoreactivity in a polymorphic bi-component solid through large synthons
Source: Commun Chem. 2025 Apr 30;8:130. doi: 10.1038/s42004-025-01527-w (PMC12043805; doi:10.1038/s42004-025-01527-w)
Supplement: Supplementary file 2 — Description of Additional Supplementary Files [file 42004_2025_1527_MOESM2_ESM.pdf]

# Description of Additional Supplementary Files

**File name: Supplementary Data 1**

**Description:** Bza.1-Form I cif file

**File name: Supplementary Data 2**

**Description:** Bza.1-Form II cif file

**File name: Supplementary Data 3**

**Description:** NMR spectra
