# Supplementary material for: Regulating photoreactivity in a polymorphic bi-component solid through large synthons
Source: Commun Chem. 2025 Apr 30;8:130. doi: 10.1038/s42004-025-01527-w (PMC12043805; doi:10.1038/s42004-025-01527-w)

**(1) Bza-1-Form I before Irradiation-^1^H-CDCl_3_-400 MHz:**

**
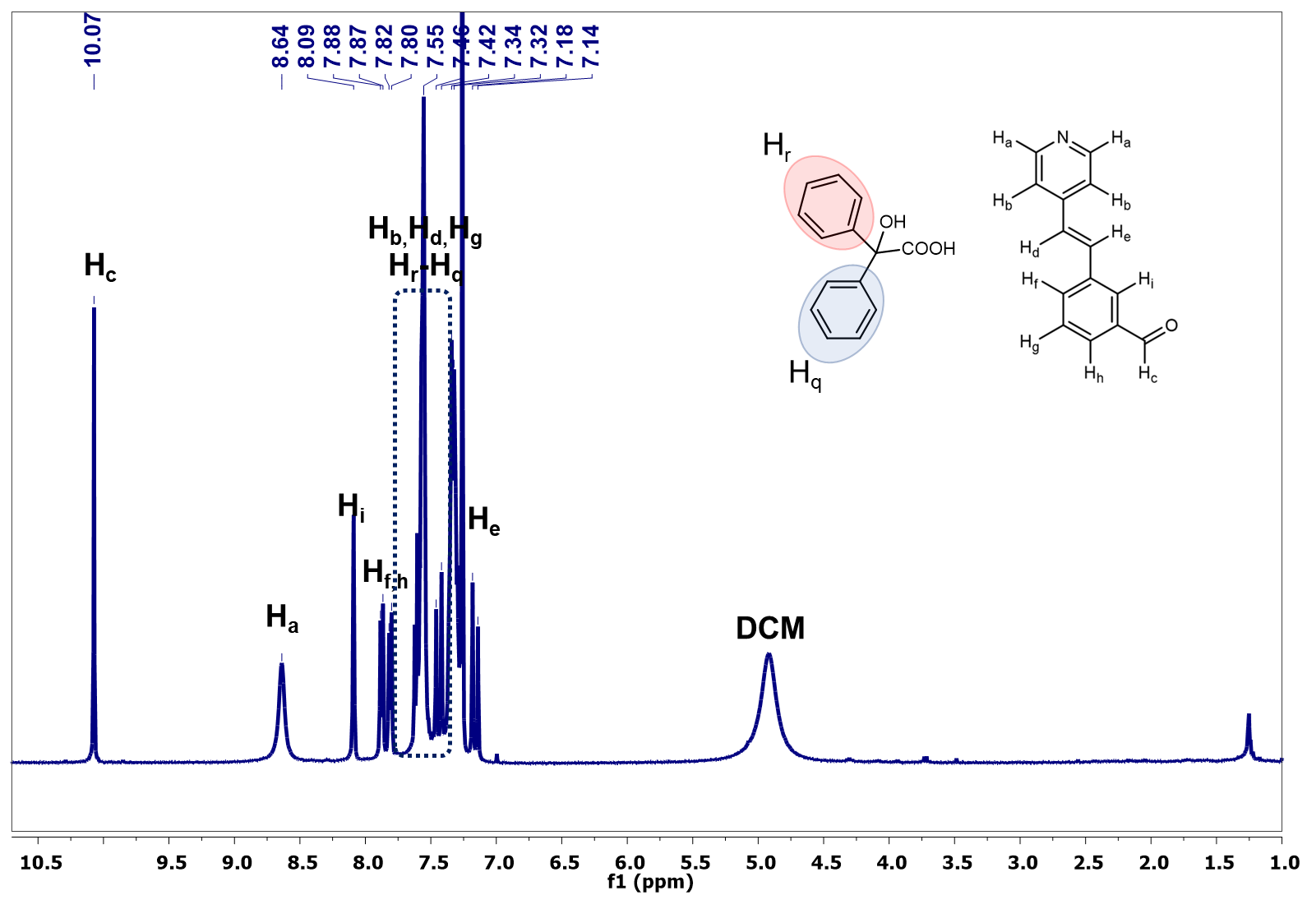
**

**(2) Bza-1-Form I after Irradiation (12 hours) ^1^H-CDCl_3_-400 MHz:**


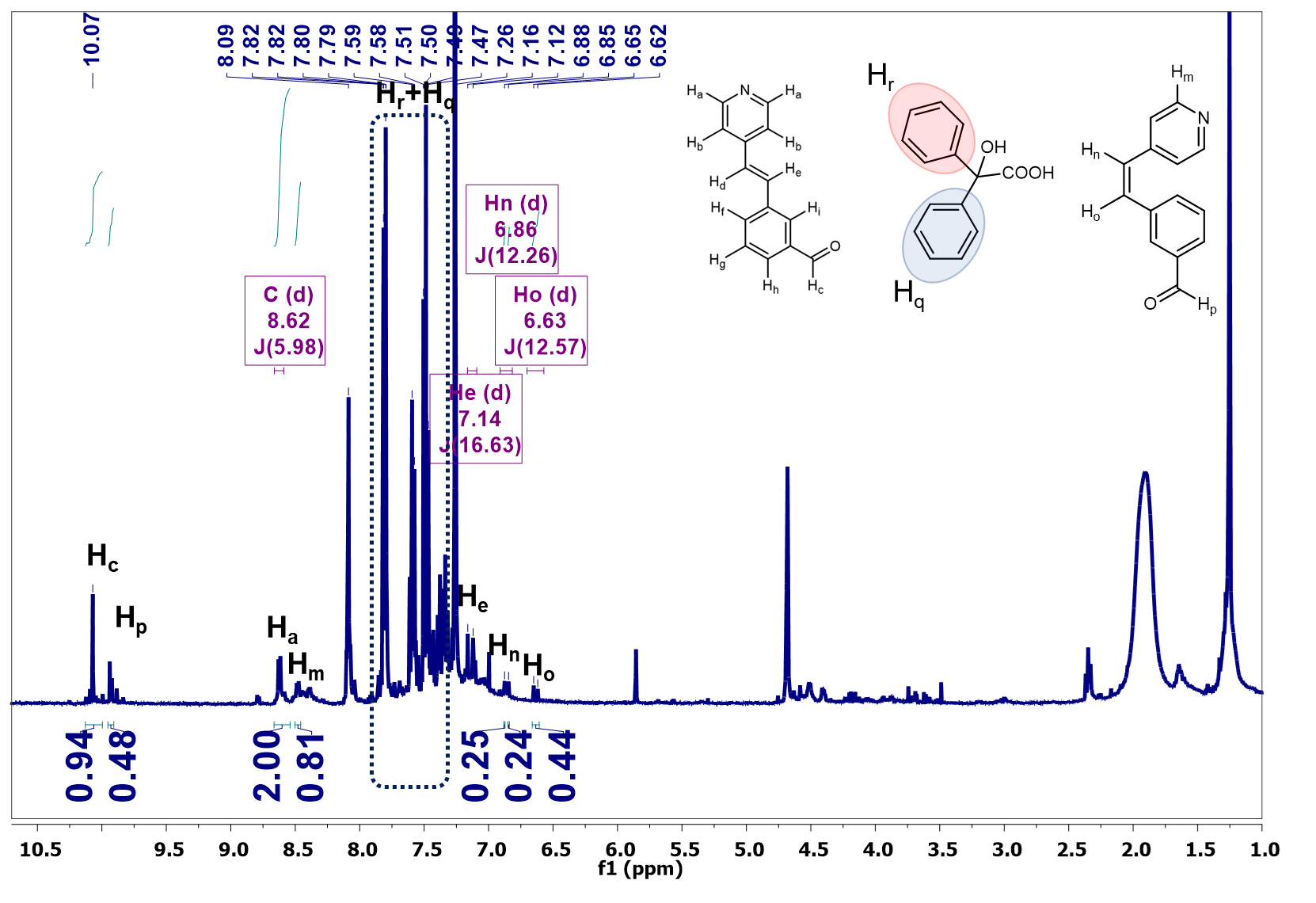


**(3) Bza-1-Form II before Irradiation-^1^H-CDCl_3_-400 MHz:**


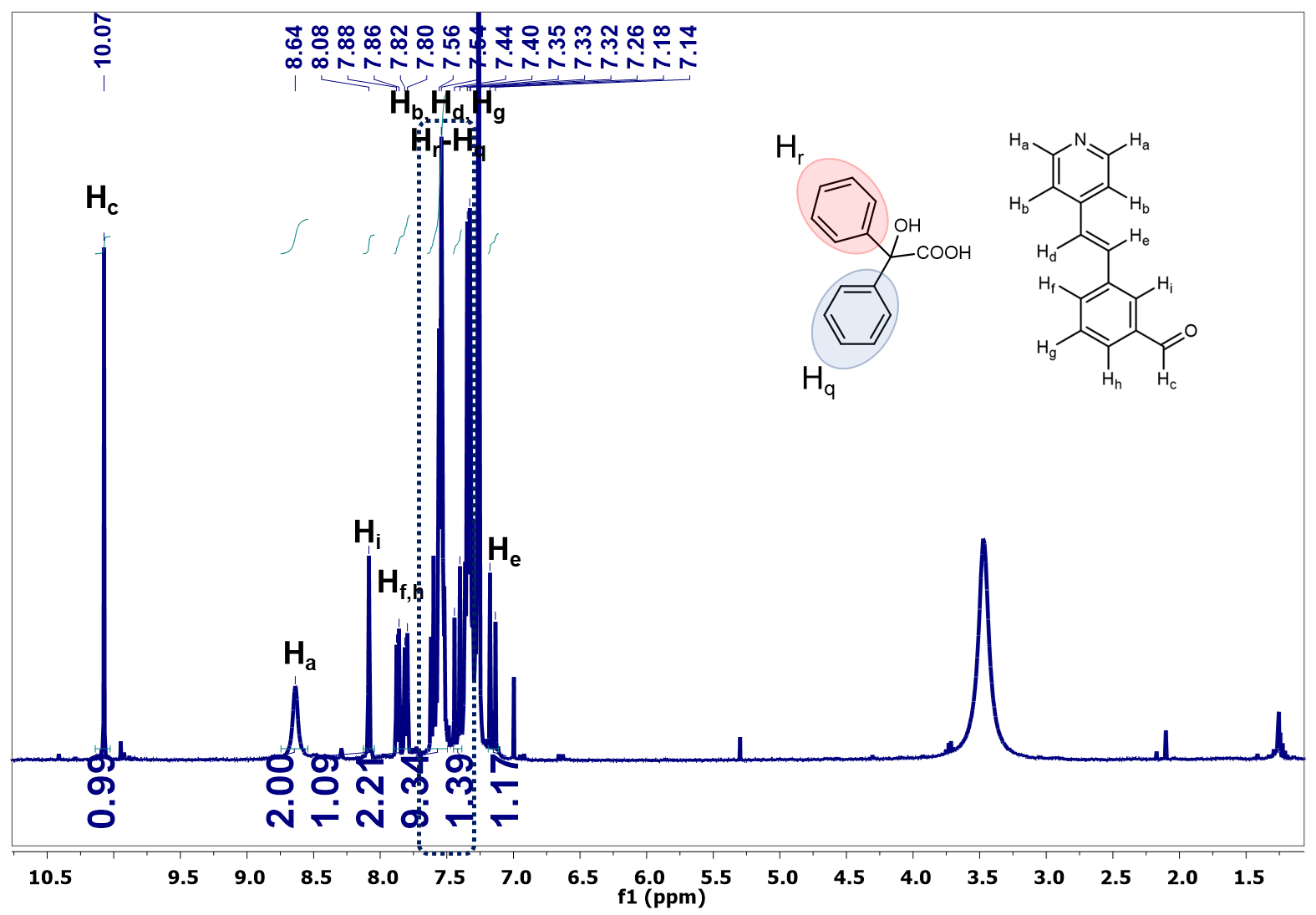


**(4) Bza-1-Form II after Irradiation (12 hours) ^1^H-CDCl_3_-400 MHz::**


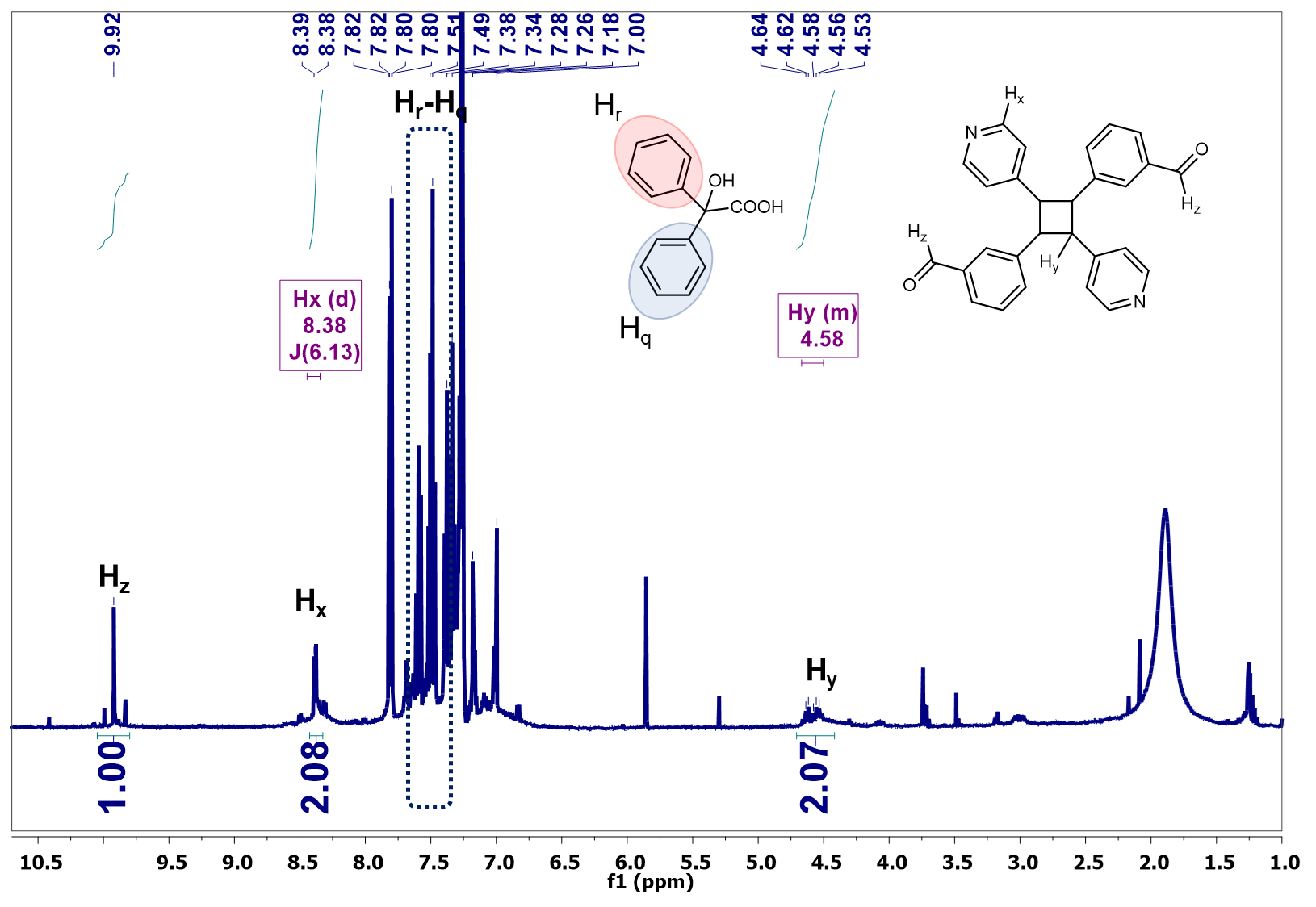

Supplement: Supplementary file 5 — Supplementary Data 3 [file 42004_2025_1527_MOESM5_ESM.docx]
